# Hsa_circ_0058495-mediated IGF2BP2 ubiquitination and m6A modification of MEKK1 promote the progression of PDAC

**DOI:** 10.7150/thno.117202

**Published:** 2025-09-12

**Authors:** Shengnan Lv, Jian Zhang, Xinyu Peng, Huan Liu, Tongjia Chu, Ziyu Liu, Kehang Duan, Jianxiong Guo, Jie Wang, Yan Liu, Feng Wei

**Affiliations:** 1 Department of Hepatobiliary and Pancreatic Surgery, General Surgery Center, The First Hospital of Jilin University, Changchun, Jilin, China, 130021.; 2 Key Laboratory of Jilin Province for Zoonosis Prevention and Control, Changchun Veterinary Research Institute, Chinese Academy of Agricultural Sciences, Jilin, China, 130117.

**Keywords:** PDAC, circRNA, ubiquitination, m6A modification, M2 polarization

## Abstract

**Background**: Pancreatic ductal adenocarcinoma (PDAC) is a highly aggressive malignancy with dismal clinical outcomes. We identified hsa_circ_0058495 as significantly upregulated in PDAC tissues and PDAC cell-derived exosomes, where it contributes to tumor proliferation and invasion. The molecular mechanisms underlying its oncogenic function, however, remain incompletely understood.

**Methods:** Differential circRNA expression was profiled by RNA sequencing. The functional role of hsa_circ_0058495 and its molecular interactions were interrogated through Western blotting, RT-qPCR, co-immunoprecipitation, RNA pull-down, and RNA immunoprecipitation assays. Confocal microscopy and PET/CT imaging were employed to delineate its biological effects *in vitro* and *in vivo*.

**Results:** Hsa_circ_0058495 was enriched in PDAC-derived exosomes and stabilized IGF2BP2 by preventing TRIM25-mediated ubiquitination and attenuating autophagy-dependent degradation. Stabilized IGF2BP2 enhanced the stability of MEKK1 mRNA, leading to sustained ERK1/2 phosphorylation and consequent promotion of PDAC cell proliferation and invasion. Moreover, exosomal hsa_circ_0058495 facilitated M2 macrophage polarization, thereby fostering an immunosuppressive tumor microenvironment.

**Conclusions:** Hsa_circ_0058495 promotes PDAC progression by stabilizing IGF2BP2 and activating the MEKK1-ERK signaling cascade, while exosomal transfer of hsa_circ_0058495 drives M2 macrophage polarization to reinforce tumor-associated immunosuppression. These findings establish hsa_circ_0058495 as a pivotal regulator of PDAC progression and underscore its potential utility as both a diagnostic biomarker and a therapeutic target.

## Introduction

Pancreatic ductal adenocarcinoma (PDAC) is a prevalent and highly aggressive malignancy, characterized by an alarmingly low 5-year survival rate of just 9% 1. Although neoadjuvant therapies, particularly FOLFIRINOX, have improved patient outcomes compared to conventional chemotherapy, the development of drug resistance remains a formidable obstacle in the treatment of PDAC 2. Therefore, identifying novel molecular targets remains a critical avenue for enhancing therapeutic efficacy and patient prognosis.

Circular RNAs (circRNAs) are a distinct class of non-coding RNAs that arise from back-splicing of pre-messenger RNA (pre-mRNA), and are ubiquitously expressed across a wide range of species, from viruses to mammals. To date, tens of thousands of circRNAs have been identified in humans, many of which have been implicated in various diseases, including viral infections and metabolic disorders. Particularly, circRNAs in cancer have garnered considerable attention due to their involvement in tumorigenesis through diverse molecular mechanisms. For instance, circPVT1 serves as a ceRNA to sponge miR-181a-2-3p, thus stabilizing ESR1 mRNA and promoting the genesis of estrogen receptor (ER)-positive breast cancer 3. In bladder cancer, circXRN2 interacts with LATS1, preventing its degradation via SPOP 4. Furthermore, circRNAs packaged into exosomes can influence the tumor microenvironment (TME). Exosomal circTRPS1 derived from bladder cancer has been shown to promote CD8^+^ T cell exhaustion by sponging miR-141-3p and upregulating GLS1, which inhibits GSH production 5. In gastric cancer, exosomal circATP8A1 enhances M2 macrophage polarization by binding miR-1-3p, thus activating the STAT6 pathway 6. Despite these insights, the functional roles and mechanisms by which circRNAs contribute to PDAC initiation and progression remain inadequately elucidated.

Insulin-like growth factor 2 mRNA-binding protein 2 (IGF2BP2) is a pivotal RNA-binding protein (RBP) that plays a significant role in tumorigenesis and the creation of an immunosuppressive microenvironment 7, 8. Aberrant expression and activity of IGF2BP2 has been documented across multiple malignancies, underscoring its oncogenic potential. The proteostasis of IGF2BP2 is largely governed by post-translational modifications (PTMs), with ubiquitination representing the most extensively characterized mechanism. In colorectal cancer and lung cancer, the E3 ubiquitin ligases TRIM21 and FBW7 have been reported to mediate ubiquitin-dependent proteasomal degradation of IGF2BP2 9, 10. Recent evidence suggests that noncoding RNAs contribute to IGF2BP2 stabilization by interfering with its ubiquitination. For instance, circEZH2, lncRNA LINRIS, and circNDUFB2 have been shown to physically associate with IGF2BP2, thereby shielding it from ubiquitin-mediated turnover 11-13. Moreover, as a well-known m6A reader, the effects of IGF2BP2 are mediated via m6A-dependent or independent pathways. For example, IGF2BP2 stabilizes LDHA mRNA by interacting with circARHGAP19, thus promoting prostate cancer by enhancing glycolysis 14. In colorectal carcinoma (CRC), IGF2BP2 binds the 3' untranslated region (3' UTR) of HMGA1, augmenting its stability and driving tumor growth and metastasis 15. In addition, IGF2BP2 has been reported to enhance CDK6 translation by directly binding to m6A-modified mRNA, which promotes the progression of triple-negative breast cancer (TNBC) 16. Moreover, IGF2BP2 has been shown to reinforce MYC oncogenic signaling by binding methylated coding region instability determinant (CRD) elements, stabilizing MYC mRNA, and enhancing its translation 17.

Our study reveals that hsa_circ_0058495 plays a crucial role in PDAC tumorigenesis and progression. We found that hsa_circ_0058495 directly interacts with IGF2BP2, suppressing its ubiquitination-mediated degradation by TRIM25. This interaction enables IGF2BP2 to promote tumor progression by stabilizing MEKK1 mRNA in an m6A-dependent manner and activating the p-ERK1/2 pathway. Notably, exosomal hsa_circ_0058495 also positively regulates M2 macrophage polarization, further contributing to PDAC development. Our findings highlight the essential role of hsa_circ_0058495 in PDAC and identify it as a potential therapeutic target for this devastating disease.

## Results

### Hsa_circ_0058495 is upregulated in PDAC

**Figure 1:**
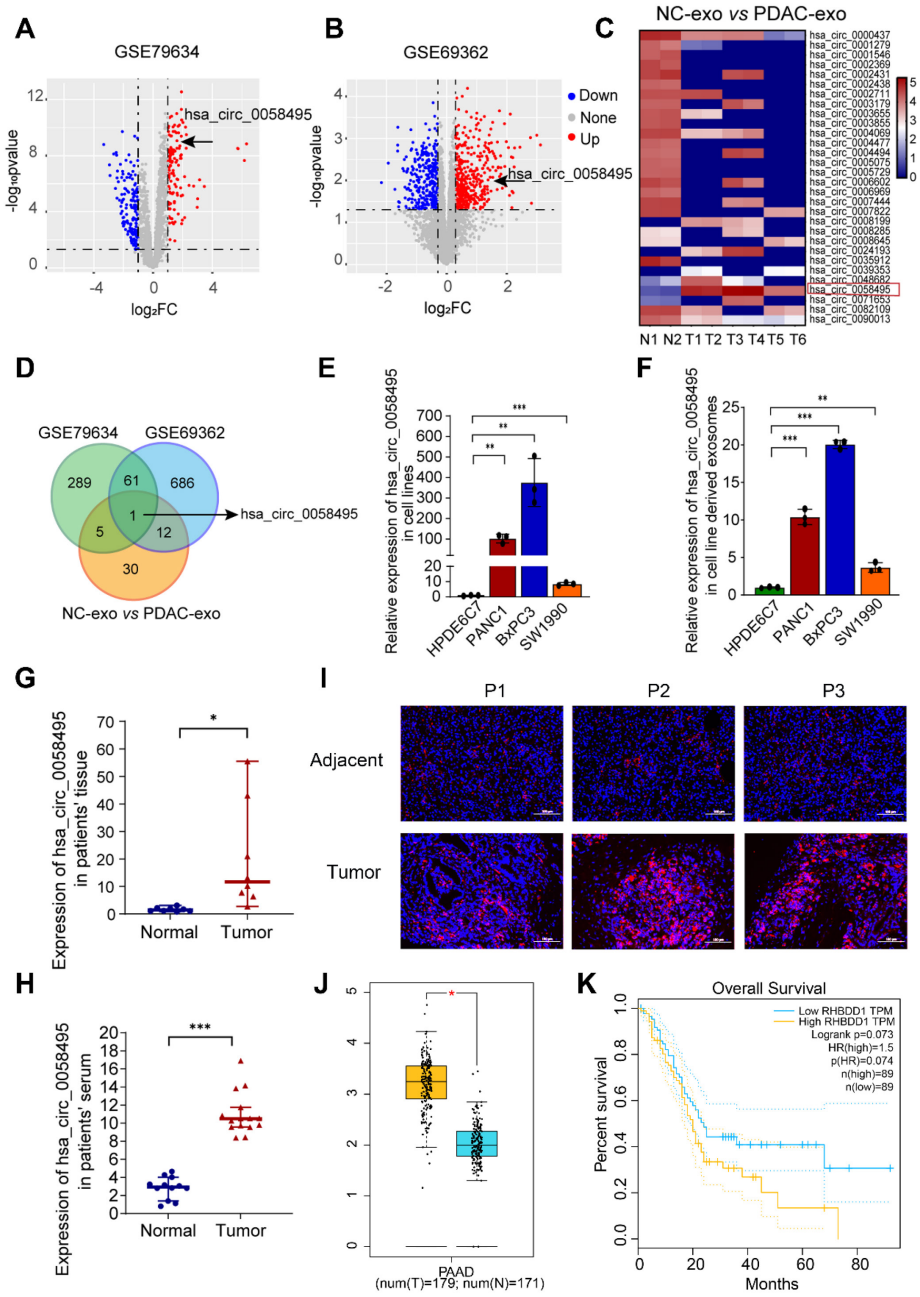
** Hsa_circ_0058495 is upregulated in PDAC.** (A-B) Volcano plots of circRNAs differentially expressed in tumor and adjacent tissues in PDAC of GSE79634 and GSE69362 datasets. (C) Heatmap showing the levels of circRNAs in PDAC tumor and normal tissues derived exosomes detected by high-throughput sequencing. (D) Venn diagram showing the overlapping circRNAs in PDAC tissues and exosomes. (E-F) Results of RT-qPCR showing the level of hsa_circ_0058495 in HPDE6C7, PANC1, BxPC3, and SW1990 cells and cell-derived exosomes. (G-H) Results of RT-qPCR showing the level of hsa_circ_0058495 in tumor and adjacent tissues and serum from PDAC patients and healthy individuals. (I) Results of FISH assay showing the abundance of hsa_circ_0058495 in PDAC tissues and adjacent tissues. Hsa_circ_0058495 (red) and nuclei (blue) were shown. Scale bar, 100 µm. (J) Boxplot showing the level of RHBDD1 mRNA in the tumor and normal tissues of PDAC in TCGA datasets. (K) Kaplan-Meier survival analysis showing the correlation between the level of RHBDD1 mRNA and patient's overall survival time of PDAC in TCGA datasets. ns, no significant; *P < 0.05; **P < 0.01; ***P < 0.001; ****P < 0.0001.

To explore the role of circRNAs in PDAC, we analyzed the expression profiles of circRNAs in PDAC and adjacent non-cancerous tissues from Gene Expression Omnibus (GEO) database. A total of 289 and 686 differentially expressed circRNAs were identified in the GSE79634 and GSE69362 datasets, respectively (log_2_^FC^ > 1.2 and p.adj < 0.05) (Figure 1A-B and Table S1). Based on the observation that circRNAs can be transferred *via* exosomes within the TME, we performed exosomal circRNA sequencing and 30 differentially expressed circRNAs were identified from PDAC tissues-derived exosomes in comparison to normal tissue-exosomes (Figure 1C and Table S2). These circRNAs from GSE79634, GSE69362 and exosome sequencing were overlapped, among which only hsa_circ_0058495 (circRHBDD1) was identified (Figure 1D). Subsequently, we verified the expression of hsa_circ_0058495 in PDAC cell lines and the result demonstrated that it was upregulated in BxPC3, PANC1, and SW1990 compared to HPDE6C7, as well as paired exosomes by RT-qPCR (Figure 1E-F). We then validated the upregulation of hsa_circ_0058495 in clinical PDAC tissues and patient serum samples (Figure 1G-H). In addition, the fluorescence *in situ* hybridization (FISH) results also demonstrated more abundant expression of hsa_circ_0058495 in PDAC tissues compared to adjacent non-cancerous tissues (Figure 1I). We collected the follow-up data of these patients and performed the Kaplan-Meier survival analysis to assess the correlation between hsa_circ_0058495 levels and overall survival (OS). The log-rank (Mantel-Cox) test was used to compare survival curves between groups stratified by circRNA expression (high *vs.* low). A hazard ratio (HR) of 2.411 (95% confidence interval [CI]: 1.228 - 4.736; p = 0.0024) indicated that high hsa_circ_0058495 expression was significantly associated with shorter OS in PDAC patients (Figure S1A). Then we analyzed the expression of RHBDD1 mRNA in TCGA datasets and found that RHBDD1 was higher in tumor tissues than normal tissues in PDAC (Figure 1J). However, the Kaplan-Meier survival analysis showed that the level of RHBDD1 was not significantly related to OS (p = 0.073, Figure 1K). Data from our PDAC patient cohort also showed that the level of RHBDD1 mRNA was not significantly associated with OS in (HR = 0.8938, 95% CI: 0.4682 - 1.706, p = 0.9155), consistent with the TCGA data (Figure S1B). These findings demonstrated significant upregulation of hsa_circ_0058495 in PDAC tissues and derived exosomes.

To investigate the clinical relevance of hsa_circ_0058495 in PDAC, we performed a comprehensive clinicopathological analysis using tissue specimens from 70 PDAC patients. Based on quantitative expression profiling, the cohort was stratified into two groups: high hsa_circ_0058495 expression (n = 35) and low hsa_circ_0058495 expression (n = 35). Statistical analysis revealed significant associations between elevated hsa_circ_0058495 expression and several aggressive pathological features, including vascular invasion (p = 0.013), perineural invasion (p = 0.026), advanced T classification (p = 0.001), lymph node metastasis (p = 0.004), and higher TNM stage (p = 0.038). However, no significant correlations were observed between hsa_circ_0058495 expression levels and demographic parameters (age, gender) or tumor characteristics (anatomical location, histological differentiation grade) (Table 1). These findings suggested that hsa_circ_0058495 may serve as a potential biomarker for tumor progression and metastasis PDAC.

### Hsa_circ_0058495 interacts with IGF2BP2 and upregulates the expression of IGF2BP2

**Figure 2:**
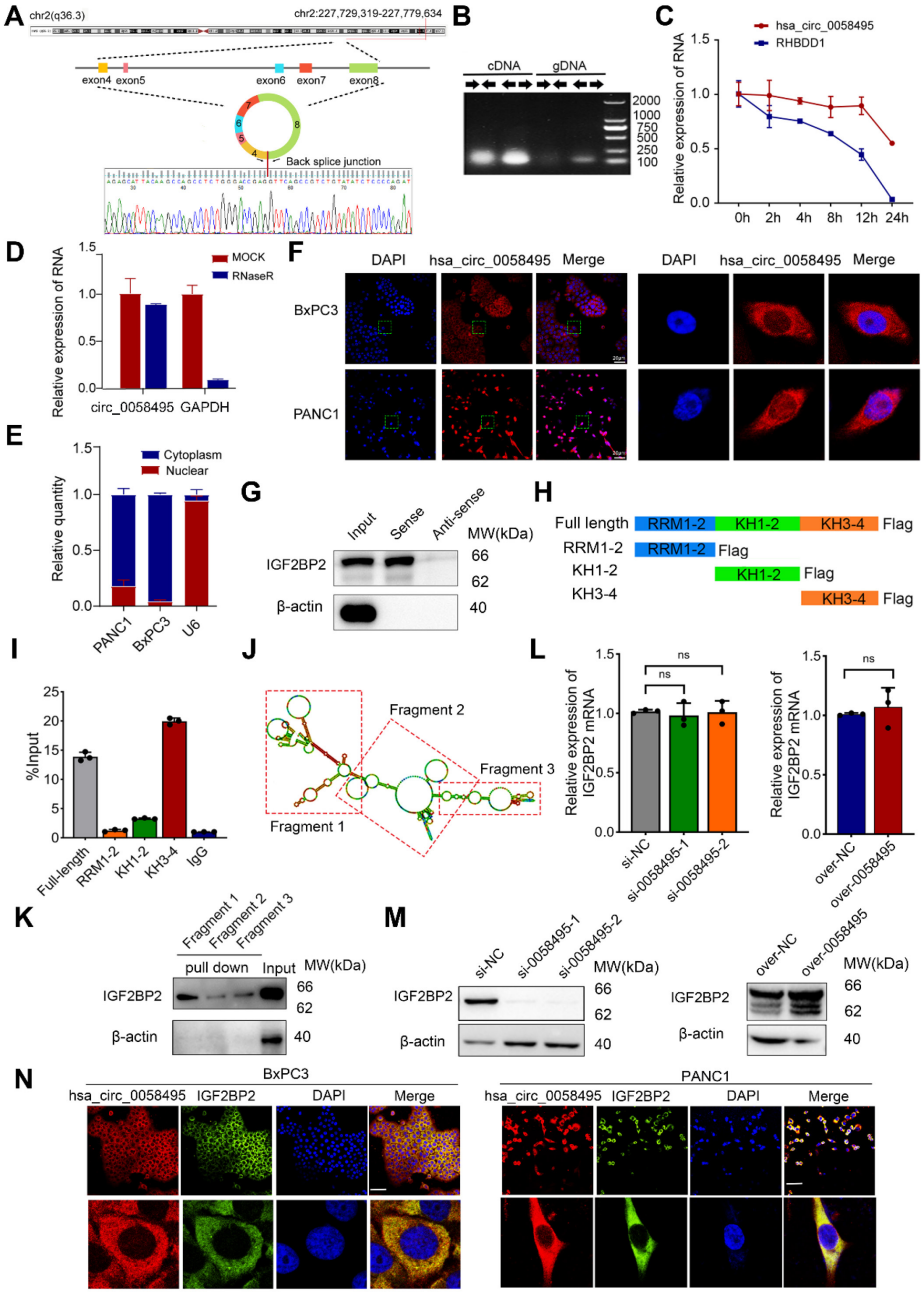
** Hsa_circ_0058495 interacts with IGF2BP2 and upregulates the expression of IGF2BP2.** (A) Schematic illustration showing the genomic localization of hsa_circ_0058495 and the back splice junction detected by Sanger sequencing. (B) Agarose gel electrophoresis analysis of PCR product amplified by divergent and convergent primers of hsa_circ_0058495 from gDNA and cDNA of BxPC3. (C) BxPC3 cells were treated with actinomycin D (5 µg/mL) for 0, 2, 4, 8, 12, and 24 hours, respectively. RT-qPCR was used to detect the level of hsa_circ_0058495 and RHBDD1 linear mRNA at the indicated times. (D) RNA was treated with RNase R (2 U/μg) at 37 ℃ for 15 minutes, and RT-qPCR was used to detect the level of hsa_circ_0058495 and GAPDH linear mRNA. (E) Nuclear-cytoplasmic fractionation RNA was extracted from BxPC3 and PANC1, and RT-qPCR was used to detect the level of hsa_circ_0058495 in nuclear and cytoplasm. (F) FISH assay showing the subcellular localization of hsa_circ_0058495 in BxPC3 and PANC1 cells. Hsa_circ_0058495 (red) and nuclei (blue) were shown. Scale bar, 20 µm. (G) RNA pull-down assay was performed by biotin-labeled hsa_circ_0058495 probe for the back splicing region and antisense probe was used as control. The level of IGF2BP2 pulled down was determined by immunoblotting, with β-actin as loading controls of input. (H) Schematic illustration of the domains contained by IGF2BP2 and shortened expressing vectors. (I) 293T cells were transfected with Flag-IGF2BP2 or Flag-RRM1-2, Flag-KH1-2, Flag-KH3-4, the cell lysates were analyzed by RNA immunoprecipitation (with anti-Flag) and RT-qPCR was used to detect the level of hsa_circ_0058495 being immunoprecipitated. (J) The secondary structure of hsa_circ_0058495 predicted by RNAfold website. (K) RNA pull-down assay was performed by biotin-labeled probes of three fragments of hsa_circ_0058495. The level of IGF2BP2 pulled down was determined by immunoblotting, with β-actin as loading control of input. (L-M) BxPC3 cells were transfected with siRNA against hsa_circ_0058495 or plasmid expressing hsa_circ_0058495 for 24 hours. The level of IGF2BP2 mRNA was detected by RT-qPCR, and the expression of IGF2BP2 protein was detected by immunoblotting. (N) Fluorescence *in situ* hybridization (FISH) by cy3-labeled hsa_circ_0058495 probe and immunofluorescence (IF) by anti-IGF2BP2 were performed in BxPC3 cells. Hsa_circ_0058495 (red), IGF2BP2 (green), and nuclei (blue) were shown. Scale bar, 20 µm. ns, no significant; *P < 0.05; **P < 0.01; ***P < 0.001; ****P < 0.0001.

Hsa_circ_0058495 is a circular non-coding RNA that originates from exons 4-8 of the *RHBDD1* gene located on chromosome 2. According to the circBase (http://www.circbase.org) and circinteractome (https://circinteractome.nia.nih.gov/) databases, hsa_circ_0058495 has a length of 946 base pairs (Figure S1C). The back-splicing site of hsa_circ_0058495 was confirmed by Sanger Sequencing (Figure 2A). The PCR analysis determined that hsa_circ_0058495 could be amplified only by divergent primers using cDNA as a template, but not with gDNA (Figure 2B). To assess RNA stability, we treated cells with actinomycin D (ActD) and found that hsa_circ_0058495 was more stable than linear RHBDD1 which was significantly degraded (Figure 2C). Moreover, hsa_circ_0058495 was validated to be resistant to RNase R exonuclease that digested linear RNAs (Figure 2D), which further confirmed its closed-loop structure of hsa_circ_0058495. Subsequently, nuclear-cytoplasmic fractionation and FISH demonstrated that hsa_circ_0058495 was mainly localized in the cytoplasm of PDAC cells (Figure 2E-F). These results confirmed that hsa_circ_0058495 is a bona fide circRNA, predominantly localizing in the cytoplasm.

CircRNAs are known to exert their functional effects through protein interactions and the regulation of complex protein modifications. To elucidate the protein-hsa_circ_0058495 interactome, we employed an RNA pull-down and mass spectrometry (MS) proteomics analysis. This approach identified 350 proteins that interacted with hsa_circ_0058495. Notably, IGF2BP2 was the only protein that overlapped with the hsa_circ_0058495-binding proteins predicted by circinteractome (Table S3). We validated this result using RNA pull-down and Western blot assays (Figure 2G). To further map the critical domain mediating the hsa_circ_0058495-IGF2BP2 association, we analyzed the IGF2BP2 protein structure using Uniprot (https://www.uniprot.org) and found that it contains two RNA recognition motifs (RRM) and four K homology (KH) domains (Figure S1D). To identify the specific domains of IGF2BP2 responsible for its interaction with hsa_circ_0058495, we constructed truncated vectors of IGF2BP2 (Figure 2H). RNA immunoprecipitation (RIP) and RT-qPCR analysis revealed that the KH3-4 domains of IGF2BP2 mediated its interaction with hsa_circ_0058495 (Figure 2I).

To further confirm this interaction, RNA pull-down assay was performed using biotin-labeled hsa_circ_0058495 truncated probes generated by T7 *in vitro* transcription. Based on the predicted secondary structure of hsa_circ_0058495 using RNAfold (http://rna.tbi.univie.ac.at//cgi-bin/RNAWebSuite/RNAfold.cgi), hsa_circ_0058495 was divided into three fragments (Figure 2J). The results showed that IGF2BP2 specifically bound to fragment 1 of hsa_circ_0058495 (Figure 2K). To elucidate the effect of hsa_circ_0058495 on IGF2BP2 protein modification, we investigated the protein and mRNA levels of IGF2BP2 in response to hsa_circ_0058495 overexpression or knockdown. The results showed that hsa_circ_0058495 upregulated the protein level of IGF2BP2 without affecting its mRNA levels (Figure 2L-M). This suggested that hsa_circ_0058495 post-transcriptionally upregulated IGF2BP2 by protecting it from degradation, rather than enhancing its transcription. Subsequently, RNA FISH-immunofluorescence data demonstrated colocalization of hsa_circ_0058495 and IGF2BP2, primarily in the cytoplasm of BxPC3 and PANC1 cells (Figure 2N). To address the subcellular localization of IGF2BP2, we conducted cytoplasmic and nuclear protein fractionation experiments and the Western blot results revealed that IGF2BP2 was predominantly localized in the cytoplasm, exhibiting a cytoplasmic-to-nuclear abundance ratio of approximately 9:1 (Figure S1E). In conclusion, our findings demonstrated that hsa_circ_0058495 directly interacts with and upregulates IGF2BP2.

### Hsa_circ_0058495 stabilizes IGF2BP2 protein by inhibiting autophagy-lysosome mediated degradation

**Figure 3:**
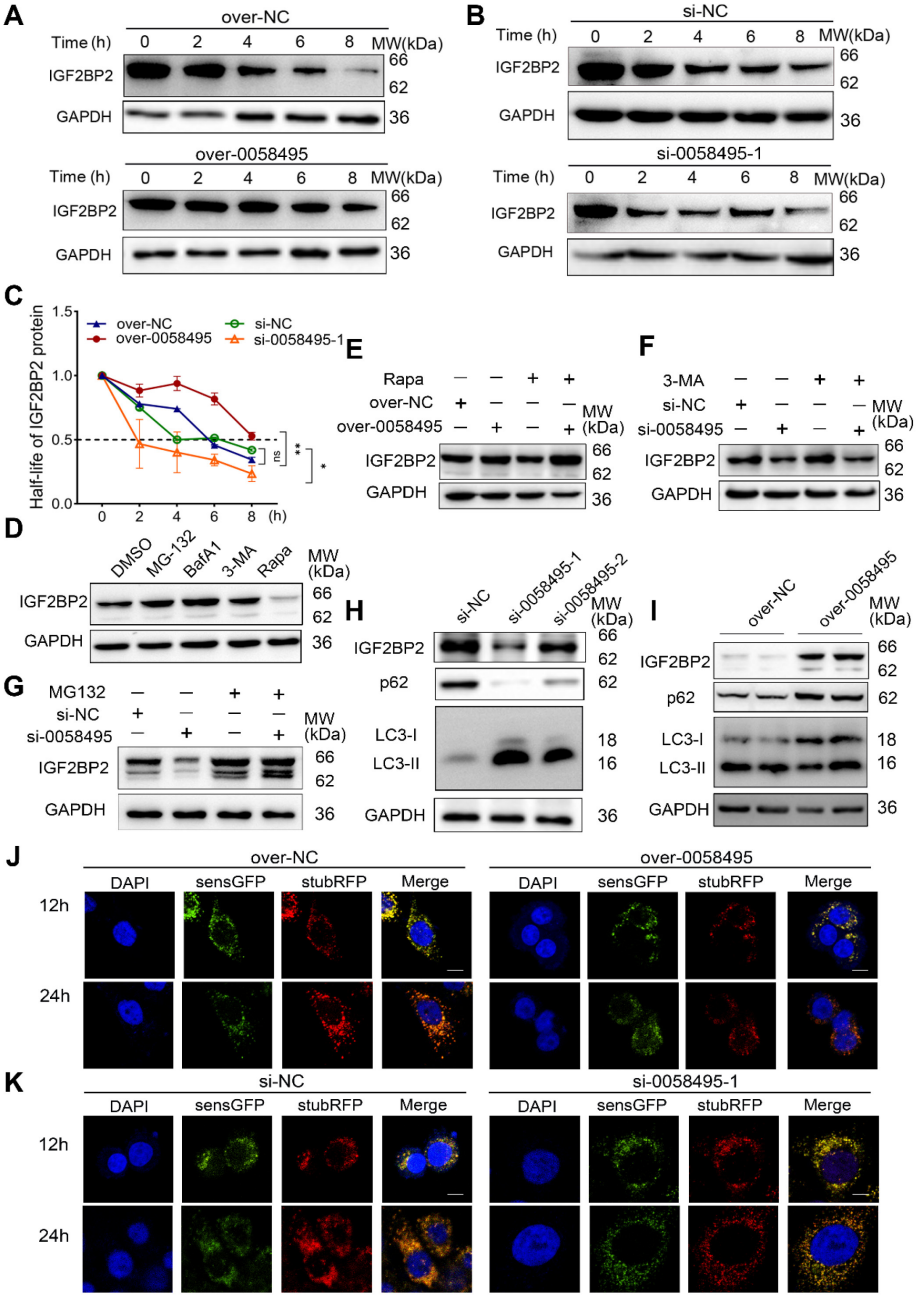
** Hsa_circ_0058495 stabilizes IGF2BP2 protein from both autophagy-lysosome and ubiquitin-proteasome mediated degradation.** (A-C) BxPC3 cells were transfected with plasmid expressing hsa_circ_0058495 or empty vector, siRNA against hsa_circ_0058495 or si-NC (negative control) for 24 hours. BxPC3 cells were treated with cycloheximide (CHX) (40 μg/mL) for 0, 2, 4, 6, and 8 hours. Subsequently, IGF2BP2 protein level was determined by immunoblotting, and the half-life of IGF2BP2 protein was calculated. (D) BxPC3 cells were treated with MG132 (10 μM), Bafilomycin A1 (BafA1, 1 μM), 3-methyladenine (3-MA, 5 mM) for 12 hours, or rapamycin (Rapa, 1 μM) for 4 hours. IGF2BP2 protein was detected by immunoblotting, with GAPDH as loading control. (E-G) BxPC3 cells were transfected with plasmid expressing hsa_circ_0058495 or empty vector in the presence or absence of rapamycin (1 μM) for 4 hours (E), with siRNA against hsa_circ_0058495 or si-NC in the presence or absence of 3-MA (5 mM) for 24 hours (F) or MG132 (10 μM) for 12 hours (G). IGF2BP2 was determined by immunoblotting, with GAPDH as loading control. (H-I) BxPC3 cells were transfected with siRNA against hsa_circ_0058495 or si-NC, plasmid expressing hsa_circ_0058495 or empty vector for 24 hours. IGF2BP2, LC3I/II, and p62 proteins were determined by immunoblotting, with GAPDH as loading controls. (J-K) BxPC3 cells with stable transfection of RFP-GFP-LC3 lentivirus were then transfected with plasmid expressing hsa_circ_0058495 or empty vector, siRNA against hsa_circ_0058495 or si-NC for 24 hours. Rapamycin (1 μM) was used to activate the process of autophagy and the fluorescence intensity was detected with confocal microscopy. Scale bar, 5 µm. ns, no significant; *P < 0.05; **P < 0.01; ***P < 0.001; ****P < 0.0001.

To further validate whether hsa_circ_0058495 mediated the upregulation of IGF2BP2 by suppressing its degradation, we treated cells transfected with negative control, hsa_circ_0058495 knockdown, or overexpression constructs with cycloheximide (CHX, a translation inhibitor). The results showed that hsa_circ_0058495 prolonged the half-life of IGF2BP2 protein (Figure 3A-C). To elucidate the mechanisms underlying the degradation of IGF2BP2, we treated BxPC3 cells with the ubiquitin-proteasome or autophagy-lysosome inhibitors or activators, MG132, bafilomycin A1 (Baf-A1), 3-methyladenine (3-MA) or rapamycin (Rapa) to identify the specific pathways and processes involved in IGF2BP2 turnover. The results indicated that IGF2BP2 degradation occurred through both the ubiquitin-proteasome and autophagy-lysosome pathways (Figure 3D). We then investigated whether the hsa_circ_0058495 modulated both pathways to suppress IGF2BP2 degradation. The BxPC3 cells with hsa_circ_0058495 knockdown or overexpression were treated with MG132, 3-MA or Rapa, respectively, and the results showed that hsa_circ_0058495 overexpression rescued IGF2BP2 levels that were decreased by rapamycin treatment, while hsa_circ_0058495 knockdown reduced IGF2BP2 levels that were increased by 3-MA or MG132 treatment (Figure 3E-G). This indicated that IGF2BP2 was degraded through both the ubiquitin-proteasome and autophagy-lysosome pathways. To investigate whether hsa_circ_0058495 stabilized IGF2BP2 proteins through regulating autophagy, we overexpressed or knocked down hsa_circ_0058495 in BxPC3 cells using pLC5-0058495 or si-0058495 and detected the level of the markers of autophagy, p62 and LC3II/I. The results showed that hsa_circ_0058495 significantly inhibited autophagy, as evidenced by a decreased LC3II/LC3I ratio and increased p62 levels. This inhibition of autophagy was associated with an increase in IGF2BP2 levels (Figure 3H-I). These findings further supported the notion that hsa_circ_0058495 prevented the degradation of IGF2BP2 through autophagy.

The autophagy process encompasses a series of consecutive events: initiation, nucleation, expansion, fusion, and cargo degradation. To understand how hsa_circ_0058495 regulated autophagy, we sought to pinpoint the specific step within this cascade that was suppressed by hsa_circ_0058495. During autophagic vesicle maturation, autophagosomes fused with lysosomes to form autolysosomes containing active proteases. We used an mRFP-GFP tandem fluorescent-tagged LC3 reporter to assess the abundance of autophagosomes and autolysosomes. The mRFP fluorophore was more tolerant to a decrease in pH than GFP; therefore, autophagosomes were labeled with both GFP and mRFP. After fusing with lysosomes, autolysosomes became acidic and GFP fluorescence was quenched. The results showed that hsa_circ_0058495 caused a dramatic decline in the number of both autophagosomes (mRFP^+^ GFP^+^) and autolysosomes (mRFP^+^ GFP^-^) in BxPC3 cells, demonstrating that autophagic flux was significantly suppressed (Figure 3J). However, knockdown of hsa_circ_0058495 significantly enhanced the formation of autophagosomes and autolysosomes in BxPC3 (Figure 3K). These findings demonstrated that hsa_circ_0058495 enhanced the expression of IGF2BP2 protein by increasing its stability, in part by suppressing the autophagy-lysosome pathway.

### Hsa_circ_0058495 suppressed ubiquitination degradation of IGF2BP2 protein by hindering the combination of TRIM25 with IGF2BP2

**Figure 4:**
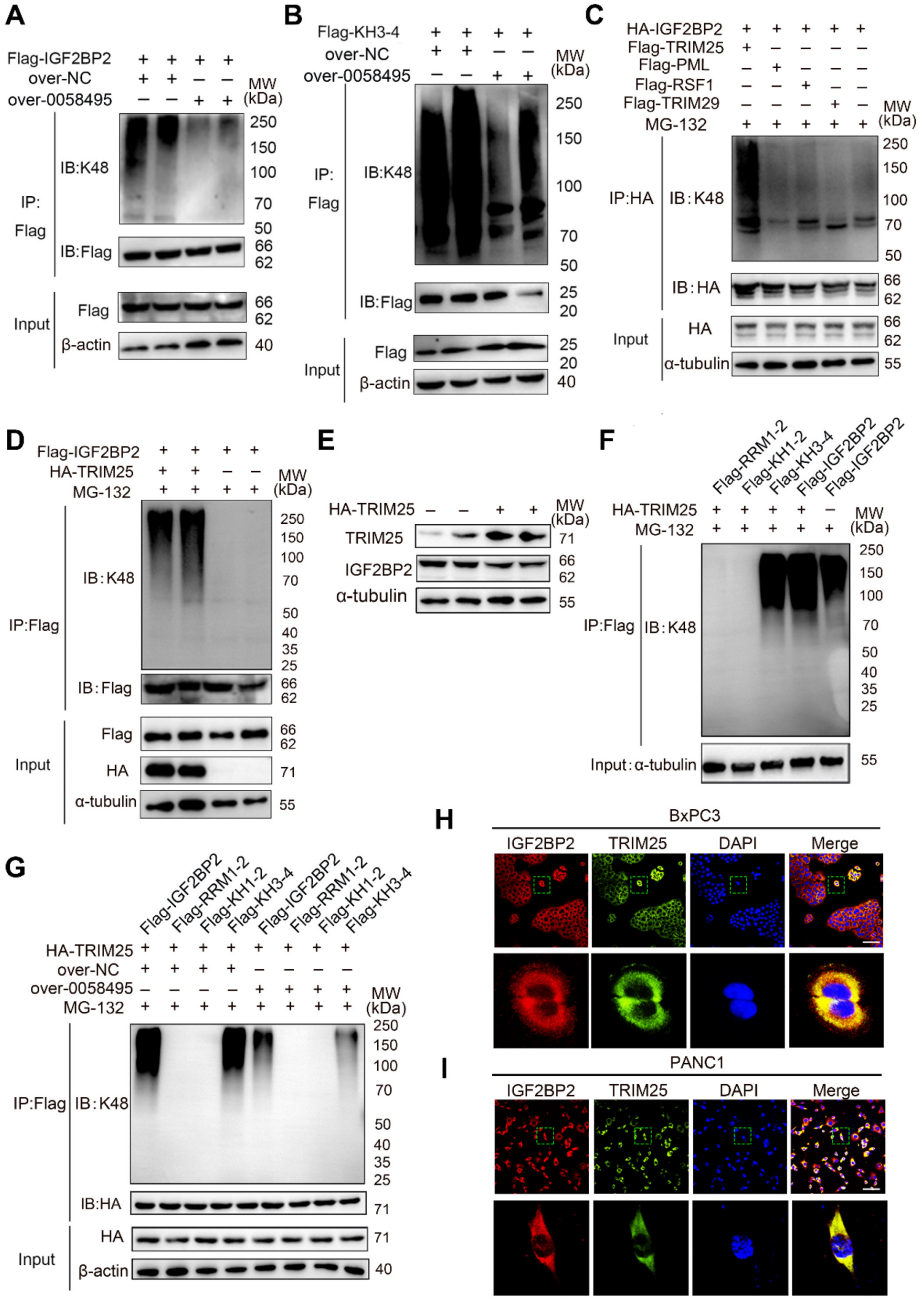
** Hsa_circ_0058495 stabilizes IGF2BP2 by hindering TRIM25-mediated ubiquitination**. (A) 293T cells were transfected with Flag-IGF2BP2 along with over-0058495 or over-NC and the cell lysates were analyzed by immunoprecipitation (with anti-Flag) and IB. (B) 293T cells were transfected with Flag-KH3-4 along with over-0058495 or over-NC and the cell lysates were analyzed by immunoprecipitation (with anti-Flag) and IB. (C) 293T cells were transfected with Flag-TRM29 or Flag-PML, Flag-RSF1, Flag-TRIM25 along with HA-IGF2BP2 and the cell lysates were analyzed by co-immunoprecipitation (with anti-HA) and IB. (D) 293T cells were transfected with Flag-IGF2BP2 and HA-TRIM25 or empty vector with HA tag. The cell lysates were analyzed by co-immunoprecipitation (with anti-Flag) and IB. (E) 293T cells were transfected with HA-TRIM25 or empty vector. IGF2BP2 and TRIM25 proteins were detected by immunoblotting, with α-tubulin as loading control. (F) 293T cells were transfected with Flag-IGF2BP2 or shortened expressing vectors Flag-RRM1-2, Flag-KH1-2, Flag-KH3-4 along with HA-TRIM25. The cell lysates were analyzed by Co-immunoprecipitation (with anti-Flag) and IB. (G) 293T cells were transfected with Flag-IGF2BP2 or shortened expressing vectors Flag-RRM1-2, Flag-KH1-2, Flag-KH3-4 along with HA-TRIM25, in the presence of over-0058495 or over-NC. The cell lysates were analyzed by co-immunoprecipitation (with anti-Flag) and IB. (H-I) Immunofluorescence (IF) assay was performed to determine the localization of IGF2BP2 and TRIM25 in BxPC3 and PANC1 cells. TRIM25 (green), IGF2BP2 (red) and nuclei (blue) are shown. Scale bar, 20 µm. (A)-(D) and (F)-(G): twenty-four hours after transfection, the cells were treated with 10 µM MG132 for 12 h before harvest.

As shown in Figure 3D and 3G, MG132, a proteasome inhibitor, also increased the level of IGF2BP2. We thus explored whether hsa_circ_0058495 also protected IGF2BP2 from ubiquitination-mediated degradation. We detected the ubiquitination level of IGF2BP2 in 293T cells overexpressing hsa_circ_0058495 and treated with MG132 by immunoprecipitation (IP) (Figure 4A). The results showed that hsa_circ_0058495 inhibited the ubiquitination-mediated degradation of IGF2BP2.

As shown in Fig. 2I, the KH3-4 domains were responsible for binding with hsa_circ_0058495. To further validate which domains of IGF2BP2 were targeted for ubiquitination inhibition by hsa_circ_0058495, we co-transfected vectors expressing truncated KH3-4 and hsa_circ_0058495 overexpression vectors into 293T cells. IP assay revealed that hsa_circ_0058495 overexpression inhibited the ubiquitination of KH3-4 domains (Figure 4B). These findings collectively demonstrated that hsa_circ_0058495 directly interacted with IGF2BP2 and protected it from degradation by ubiquitination.

To investigate whether hsa_circ_0058495 suppressed IGF2BP2 ubiquitination by competitively blocking the binding sites for E3 ligases on IGF2BP2, we performed IP- MS in 293T cells transfected with HA-IGF2BP2 or HA-NC. This analysis identified a total of 280 proteins associated with IGF2BP2, among which, four E3 ligases TRIM29, PML, RSF1, and TRIM25 were identified (Table S4). Further co-immunoprecipitation (Co-IP) assay in cells co-transfected with each of the E3 ligases and IGF2BP2 revealed that only TRIM25 significantly enhanced the ubiquitination of IGF2BP2, while the other three E3 ligases had no effect (Figure 4C-D). Consistently, Western Blot analysis showed that overexpression of TRIM25 decreased IGF2BP2 protein levels (Figure 4E).

To delineate the specific domains of IGF2BP2 that were targeted by TRIM25 for ubiquitination and that also interacted with hsa_circ_0058495, we co-transfected the indicated truncated IGF2BP2 vectors and an HA-Ub plasmid into 293T cells, which were then treated with MG132. IP analysis revealed that the KH3-4 domains were significantly ubiquitinated by TRIM25, whereas the RRM1-2 and KH1-2 domains were not (Figure 4F). To further elucidate the mechanism by which hsa_circ_0058495 suppressed IGF2BP2 ubiquitination, we performed co-IP assay to determine if hsa_circ_0058495 functions by preventing TRIM25 from interacting with IGF2BP2 through steric hindrance effect. Our results showed that the overexpression of hsa_circ_0058495 decreased the ubiquitination level of KH3-4 domains of IGF2BP2 mediated by TRIM25 (Figure 4G). Additionally, immunofluorescence (IF) assay demonstrated colocalization of TRIM25 with IGF2BP2 in the cytoplasm both in BxPC3 and PANC1 cell lines (Figure 4H-I). To summarize, these findings indicated that TRIM25 promoted the ubiquitination of IGF2BP2 predominantly on its KH3-4 domains, and that hsa_circ_0058495 significantly suppressed this process, thereby protecting IGF2BP2 protein from degradation.

### Hsa_circ_0058495 promotes the proliferation and invasion of PDAC cells *in vitro* and* in vivo*

**Figure 5:**
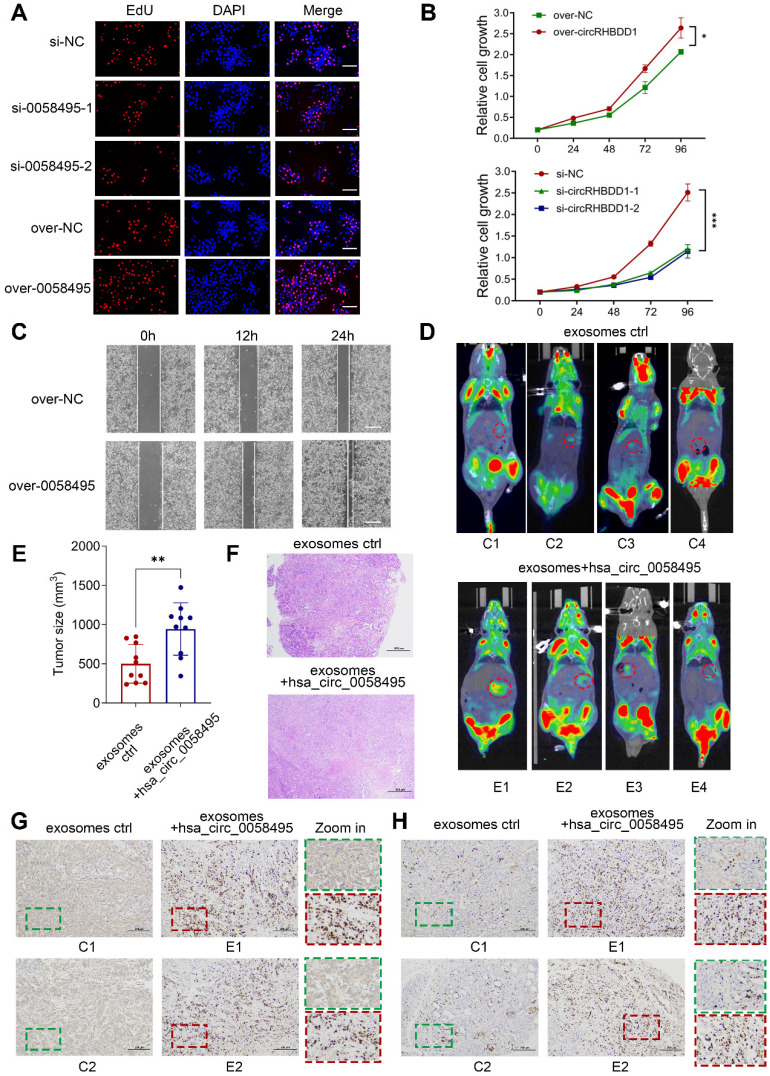
** Hsa_circ_0058495 promotes the proliferation and invasion of PDAC cells *in vitro* and *in vivo***. (A) BxPC3 cells were transfected with siRNA against hsa_circ_0058495 or plasmid expressing hsa_circ_0058495 for 24 hours. EdU assay was performed to assess the proliferation ability of cells. Proliferated cells (red) and nuclei (blue) were shown. Counts of total cells and proliferated cells are shown in the column. Scale bar, 40 µm. (B) The same transfected cells were fixed and CCK-8 assay was applied to determine the growth rate of cells. (C) The same transfected cells were fixed and wound healing assay was used to determine invasion ability. The cell invasion rate was calculated and shown in the column. Scale bar, 80 µm. (D) Imaging of PET/CT for KPC mice injected exosomes overexpressed hsa_circ_0058495 or not through tail veins. (E) Tumor sizes of KPC mice. (F) H&E staining for tumor tissues from KPC mice. Scale bar, 1000 µm. (G-H) IHC staining of IGF2BP2 and Ki67 protein for tumor tissues from KPC mice injected exosomes overexpressed hsa_circ_0058495 or not through tail veins. Scale bar, 200 µm. ns, no significant; *P < 0.05; **P < 0.01; ***P < 0.001; ****P < 0.0001.

To investigate the role of hsa_circ_0058495 in PDAC progression, we constructed siRNAs targeting hsa_circ_0058495 (si-0058495) to knockdown its expression and an over-expression plasmid (pLC5-0058495) to ectopically express hsa_circ_0058495. The efficiency of hsa_circ_0058495 knockdown and overexpression was confirmed in BxPC3 and PANC1 cells (Figure S1F). Subsequent experiments demonstrated that the capacities of PDAC cell proliferation, invasion, and migration were regulated by silencing or overexpression of hsa_circ_0058495 *in vitro*. The EdU assay showed that hsa_circ_0058495 promoted the proliferation of BxPC3 and PANC1 cells (Figure 5A and Figure S1G). The CCK-8 assay showed hsa_circ_0058495 promoted cancer cell proliferation as indicated by cell growth curve (Figure 5B). Besides, the wound healing assay determined that hsa_circ_0058495 accelerated cancer cell invasion (Figure 5C and Figure S1H).

For the* in vivo* study, we used a spontaneous PDAC model in KPC (LSL-Kras^G12D-/+^; LSL-Trp53^R172H-/+^; Pdx-1-Cre) mice and injected exosomes with or without hsa_circ_0058495 overexpression through tail veins twice a week for 6 weeks (Figure S2A). The PET-CT scanning was used to evaluate the orthotopic tumorigenicity of KPC mice and the ^18^F-fluorodeoxyglucose (^18^FDG) accumulation of the orthotopic tumors in the pancreas. The standard uptake value (SUV) of was analyzed (Table S5). The data revealed that the exosomes derived from hsa_circ_0058495-overexpressed PDAC cells significantly promoted the growth of PDAC compared with those derived from parental PDAC cells according to images shown through PET/CT (Figure 5D) and tumor sizes measurement (Figure 5E), and the H&E staining of the tumor tissues was performed (Figure 5F). Immunohistochemistry (IHC) assay of excised tumor sections showed increased expression of IGF2BP2 and Ki67 in tumors of mice treated with hsa_circ_0058495 over-expressed exosomes (Figure 5G-H). FISH assay showed that hsa_circ_0058495 was more abundant in tumor tissues isolated from mice receiving hsa_circ_0058495-overexpressing exosomes compared to the control group (Figure S2B).

In addition, to validate whether the tumor-promotion effect of hsa_circ_0058495 was correlated with the immune microenvironment, we constructed orthotopic PDAC model in immunocompetent mice (C57BL/6) and immunodeficient mice (NOD-SCID) by injecting Pan02 cells into the subcapsular region of the pancreas. Following exosomes injection from tail veins twice a week for 6 weeks, the tumors were resected and the volume and weight of tumor lesions were analyzed. H&E staining was also performed. The results showed that the tumors from the hsa_circ_0058495-overexpressing exosomes-treated groups were significantly larger than those from control group in both of immunocompetent and immunodeficient mice, suggesting that hsa_circ_0058495 promoted the orthotopic tumorigenicity of PDAC cells independently from immune status (Figure S2C-F). Consistent with this finding, NOD-SCID mice of both two groups exhibited accelerated tumor progression, attributable to compromised adaptive and innate immunity characteristic of this strain, which is characterized by the lack of functional T, B cells, deletion of complement 5, and decreased function of NK cells and macrophages. IHC analysis of Ki67 revealed that hsa_circ_0058495 overexpression facilitated the tumor growth (Figure S2G). Taken together, the results demonstrated that the function of hsa_circ_0058495 on promoting the orthotopic tumorigenicity of PDAC was not dependent on the immune status of mice.

The patient-derived xenograft (PDX) model was also conducted to further verify the effect of hsa_circ_0058495 on humanized PDAC. Patient-derived PDAC tissue blocks were implanted subcutaneously in the flank of Balb/c nude mice and the tumor sizes were recorded every 3 days. After treatment with exosomes through tail veins twice a week for 6 weeks, the tumors were resected and the volume and weight of tumor lesions were analyzed (Figure S3A-D). Then the H&E staining and IHC analysis of Ki67 were performed (Figure S3E-F). The abundance of hsa_circ_0058495 was validated by FISH assay (Figure S3G). These results showed that treatment with exosomes enriched for hsa_circ_0058495 significantly promoted the growth of humanized PDAC. Our findings collectively illustrated that hsa_circ_0058495 promoted the progression of PDAC both *in vitro* and *in vivo.*

### Hsa_circ_0058495/IGF2BP2 promotes the activation of the ERK pathway by stabilizing MEKK1 mRNA in an m6A-dependent manner

To investigate whether IGF2BP2 contributed to PDAC progression through its role as an m6A reader, we performed transcriptome sequencing of RIP samples enriched with an IGF2BP2 antibody. We also analyzed a dataset of RNA-sequencing comparing METTL3-depleted to wildtype THP-1 cells 18. We identified the overlapping molecules of these two datasets to identify differentially expressed entities after inhibiting m6A modification which could also interact with IGF2BP2 19, 20 (Table S6).

**Figure 6:**
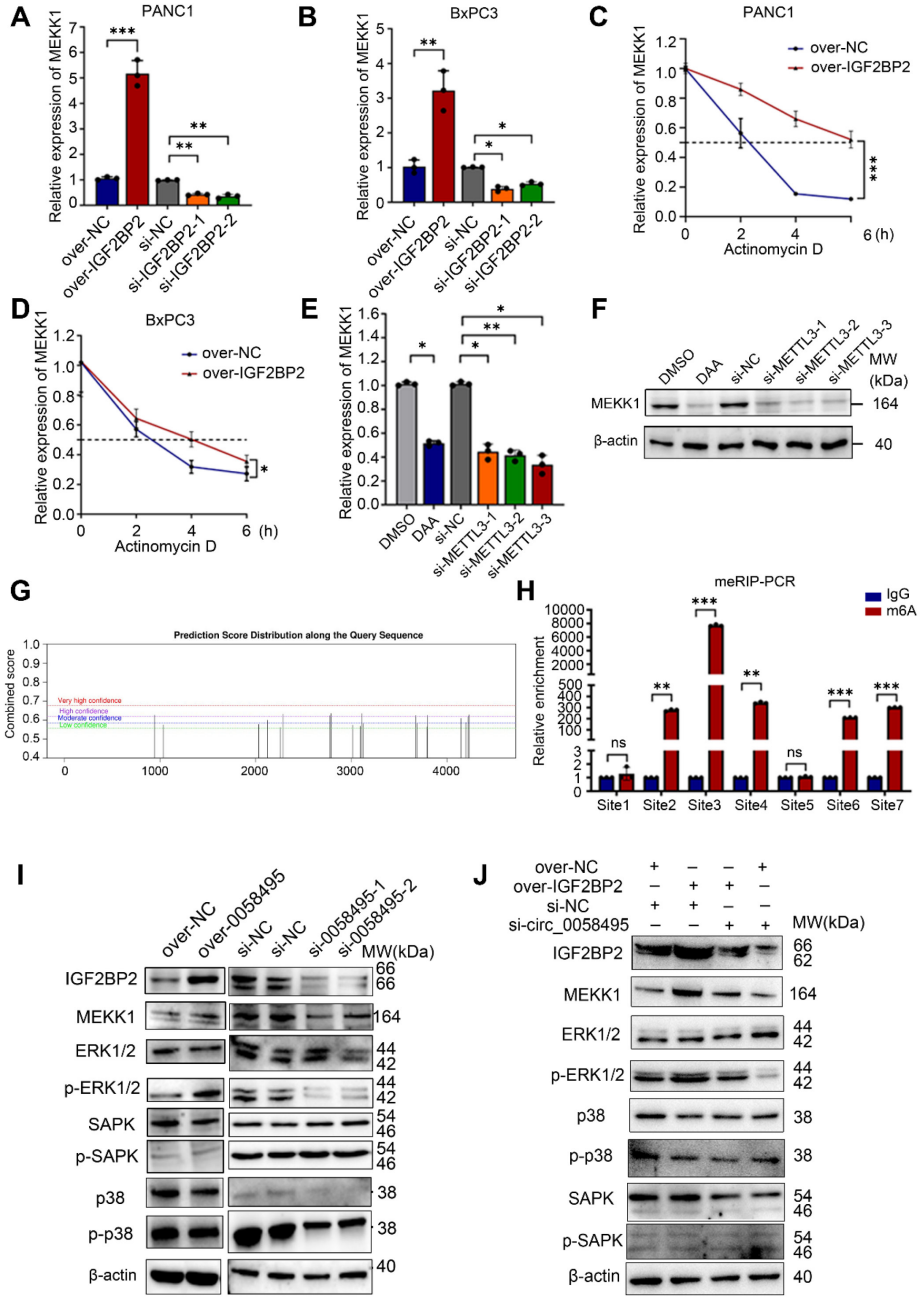
** Hsa_circ_0058495/IGF2BP2 promotes the activation of the ERK pathway by stabilizing MEKK1 mRNA in an m6A-dependent manner.** (A-B) PANC1 and BxPC3 cells were transfected with siRNA against IGF2BP2 or plasmid expressing IGF2BP2 for 24 hours. The level of MEKK1 mRNA was determined by RT-qPCR. (C-D) PANC1 and BxPC3 cells were transfected with plasmid expressing IGF2BP2 or empty vectors for 24 hours, then treated with actinomycin D (5 μg/mL) for 0, 2, 4, and 6 hours. The half-life of MEKK1 mRNA was determined by RT-qPCR. (E-F) BxPC3 cells were treated with 3-Denitroadenosine (DAA) (1 μM) or transfected with si-METTL3. The level of MEKK1 mRNA was detected by RT-qPCR; MEKK1 protein was determined by immunoblotting with β-actin as loading control. (G) The predicated m6A-modified sites on MEKK1 mRNA. (H) Methylation-RNA-immunoprecipitation (with anti-m6A) and RT-qPCR were performed to detect the enrichment of m6A modification on predicted sites. (I-J) BxPC3 cells were transfected with plasmid expressing hsa_circ_0058495 or si-0058495 for 48 hours (I) or transfected over-IGF2BP2 with or without si-0058495 for 48 hours (J), IGF2BP2, MEKK1, p-ERK1/2, ERK1/2, p-SAPK/JNK, SAPK/JNK, p-p38 and p38 proteins were determined by immunoblotting with β-actin as loading controls. ns, no significant; *P < 0.05; **P < 0.01; ***P < 0.001; ****P < 0.0001.

Finally, this analysis identified MEKK1 (also known as MAP3K1), a crucial kinase in the MAPK cascade, as the potential target of IGF2BP2. Next, to clarify the impact of IGF2BP2 on MEKK1 expression, the mRNA level of MEKK1 was detected after knockdown or overexpression of IGF2BP2. The RT-qPCR results demonstrated that IGF2BP2 positively regulated the mRNA levels of MEKK1 in both PANC1 and BxPC3 cells (Figure 6A-B). To confirm this, cells transfected with IGF2BP2-overexpressing or control plasmids were treated with Actinomycin D, and the results showed that overexpression of IGF2BP2 extended the half-life of MEKK1 mRNA by about 4 hours in PANC1 cells and 1.5 hours in BxPC3 cells (Figure 6C-D). We then investigated whether IGF2BP2 stabilized MEKK1 mRNA in an m6A-dependent manner. The m6A modification was blocked by using 3-Denitroadenosine (DAA) or knocking down METTL3 by siRNAs. As expected, suppressing m6A modification led to a significant decrease in both MEKK1 mRNA and protein levels (Figure 6E-F). To further validate that MEKK1 was modified by m6A, the SRAMP (http://www.cuilab.cn/sramp) was used to predict the potential m6A sites on MEKK1 mRNA. A total of 7 sites with high confidence were identified (Figure 6G). MeRIP-RT-qPCR assay determined that m6A modification was predominantly enriched in sites 2, 3, 4 and sites 6, 7 (Figure 6H). To further determine by which domain IGF2BP2 recognized and bound to m6A sites of MEKK1, RIP assay was performed and the results showed that KH3-4 predominantly bound to m6A sites of MEKK1 mRNA. Besides, KH1-2 had a very minor effect on binding with m6A sites and RRM1-2 showed no measurable effect (Figure S3H).

To directly establish hsa_circ_0058495's upstream regulatory role in the IGF2BP2-MEKK1 axis, the Western blot assay was firstly performed in BxPC3 cells after overexpression or knockdown of hsa_circ_0058495. The results showed that hsa_circ_0058495 promoted the expression of IGF2BP2, MEKK1 and downstream phosphorylated ERK1/2 (Figure 6I). The functional reversion experiments further confirmed that knockdown of hsa_circ_0058495 abrogated MEKK1 upregulation induced by IGF2BP2 overexpression (Figure 6J), which confirmed that hsa_circ_0058495 was the upstream regulator of this IGF2BP2/MEKK1 axis. Overall, these findings suggested that hsa_circ_0058495, by upregulating IGF2BP2, stabilized MEKK1 mRNA in an m6A-dependent manner, thereby leading to the activation of ERK1/2 pathway.

### Exosomes-delivered hsa_circ_0058495 promotes M2 polarization of macrophages

Exosomes secreted by tumor cells play a vital role in shaping the TME, in part by inducing the differentiation of immune cells. These nanovesicles facilitate the transfer of non-coding RNAs between cells, thereby modulating the immune response. As shown in Figure 1F, hsa_circ_0058495 was significantly upregulated in PDAC-derived exosomes. The multiplex immunofluorescence (mIF) assay showed that M2 macrophages were more abundant in tumors with high hsa_circ_0058495 expression (Figure S4A) and a FISH assay demonstrated that hsa_circ_0058495 was abundant in M2 macrophages in patient-derived PDAC tissues (Figure S4B). IF assay of tumor tissues from KPC mice determined that treatment by exosomes with hsa_circ_0058495 promoted M2 macrophage infiltration (Figure S4C). We investigated whether these exosomes contributed to the M2 polarization by detecting the level of Arg1 (a marker of M2) and CD86 (a marker of M1) proteins in THP-1 cells treated by exosomes from HPDE6C7, PANC1, and BxPC3 (Figure S4D). The findings showed that these exosomes promoted M2 polarization. In addition, THP-1 cells treated with exosomes showed increased levels of hsa_circ_0058495 (Figure S4E). Moreover, exosomes from PDAC cells were validated by Western blot that detecting the exosome markers, CD63 and TSG101, and transmission electron microscopy (TEM) (Figure S4F-G).

Given that IGF2BP2 was upregulated in M2 macrophages and that hsa_circ_0058495 regulated IGF2BP2, we investigated whether hsa_circ_0058495 influenced macrophage polarization after being selectively packaged into PDAC-derived exosomes. First of all, Western blot, RT-qPCR and enzyme linked immunosorbent assay (ELISA) results showed that hsa_circ_0058495 enhanced M2 macrophage polarization by detecting the markers of M1 (iNOS, IL-1β, CD86, TNF-α and CD80) and M2 (Arg1, IL-10, CD206, CCL13 and TGF-β) (Figure S5A-D). Subsequently, THP-1 cells were transfected with si-NC or si-IGF2BP2, then treated with exosomes derived from BxPC3 cells with or without hsa_circ_0058495 overexpression. The markers of M1 or M2 macrophages were detected by RT-qPCR and Western blot. We found that exosomal hsa_circ_0058495 rescued the suppression of M2 polarization induced by IGF2BP2 knockdown, suggesting that IGF2BP2 was a crucial target of hsa_circ_0058495 in inducing M2 polarization (Figure S6A-B).

Considering that IGF2BP2 was found to function by activating of the MEKK1 pathway, we further investigated the potential involvement of MEKK1 in the polarization of macrophages by transfecting with si-NC or si-MEKK1, with stimulating by exosomes derived from BxPC3 cells overexpressed hsa_circ_0058495 or not. These findings indicated that the knockdown of MEKK1 suppressed exosomal hsa_circ_0058495-induced M2 polarization (Figure S6C-D). Flow cytometry (FC) was performed to detect the macrophage polarization using CD86 as an M1 marker and CD206 as an M2 marker in tumor tissues of PDX mice models, and the results showed that the treatment of exosomes over-expressed hsa_circ_0058495 promoted the polarization of M2 macrophages whereas suppressed M1 macrophages (Figure S6E). In summary, these results demonstrated that exosomal hsa_circ_0058495 from PDAC facilitated M2 macrophage polarization by regulating IGF2BP2-MEKK1 axis in macrophages.

### Single-cell transcriptomic characterization of PDAC TME stratified by hsa_circ_0058495 expression

**Figure 7:**
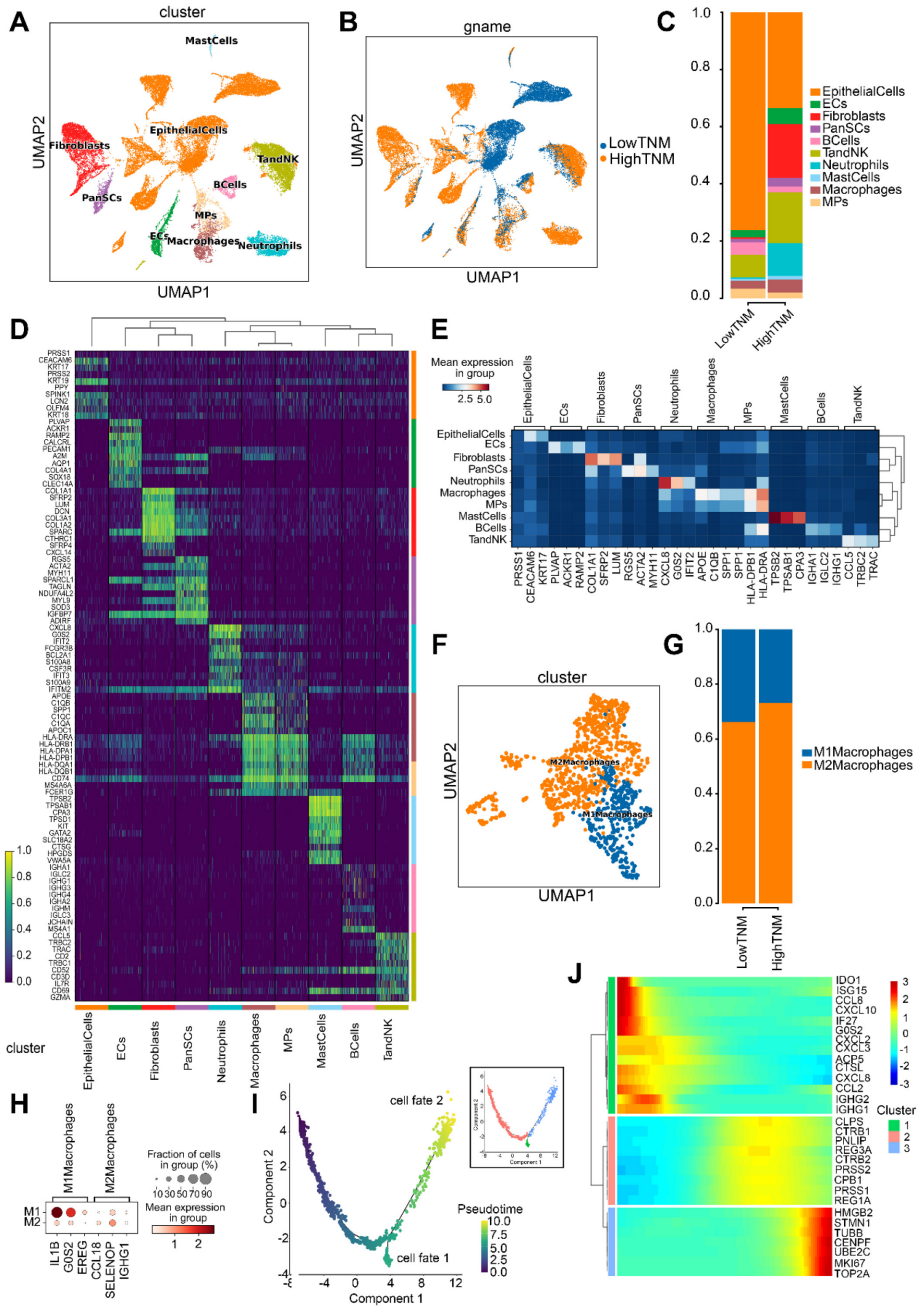
** Single-cell transcriptomic characterization of PDAC TME stratified by hsa_circ_0058495 expression.** (A) UMAP visualization of annotated cell clusters in PDAC TME. (B) UMAP projection of cellular distribution stratified by hsa_circ_0058495 expression status. (C) Cellular composition analysis comparing hsa_circ_0058495 high- and low-expression cohorts. (D) Differential expression heatmap of cluster-specific markers across cell populations. (E) Cell type-specific marker expression profile across TME compartments. (F) UMAP representation of macrophage subtypes (M1/M2) in PDAC TME. (G) Macrophage subtype distribution in hsa_circ_0058495 expression cohorts. (H) Polarization-specific marker expression profile in macrophage subtypes. (I) Pseudotemporal trajectory analysis of macrophage differentiation dynamics. (J) Expression dynamics of key regulatory genes during macrophage polarization. Color gradient represents normalized expression levels (blue: low; red: high).

To elucidate the molecular mechanisms by which hsa_circ_0058495 modulated the TME in PDAC, we conducted single-cell RNA sequencing (scRNA-seq) on five freshly resected PDAC tumor specimens obtained from distinct patients (designated P1-P5). RT-qPCR was performed to quantify hsa_circ_0058495 expression levels, which enabled us to stratify the samples into two cohorts: a low-expression group (P1 and P5) and a high-expression group (P2, P3, and P4) (Figure S7A). Following rigorous quality control measures, we successfully generated single-cell transcriptomic profiles for 31,788 cells across all specimens for subsequent bioinformatic analyses. Unsupervised clustering revealed ten distinct cellular populations representing major TME components, including epithelial cells, endothelial cells (ECs), fibroblasts, pancreatic stellate cells (PanSCs), B lymphocytes, T lymphocytes and natural killer cells (T/NK cells), neutrophils, mast cells, macrophages, and mononuclear phagocytes (MPs) (Figure 7A). The final dataset comprised 12,046 high-quality cells from the hsa_circ_0058495 low-expression cohort and 19,742 from the high-expression cohort, with proportional representation of each cellular subtype illustrated in Figure 7B-C.

Cell cluster annotation was performed through differential expression analysis to identify cluster-specific marker genes (Figure 7D). Canonical lineage markers, including *SPP1* (macrophages) 21 and *KRT19* (epithelial cells) 22, were employed for cellular classification. Additionally, we identified additional subtype-specific markers, such as *APOE* for macrophages and *KRT17* for epithelial cells (Figure 7E).

Given our previous *in vitro* findings demonstrating the role of hsa_circ_0058495 in M2 macrophage polarization, we conducted detailed characterization of the macrophage compartment within the TME. Analysis of 2,530 macrophages stratified by hsa_circ_0058495 expression status revealed significant expansion of M2 macrophages in the high-expression cohort (Figure 7F-G). Comprehensive marker analysis identified *IL1B*, *G0S2*, and *EREG* as characteristic of M1 macrophages, while *CCL18*, *SELENOP*, and *IGHG1* were established as M2 macrophage markers (Figure 7H and Figure S7B).

To delineate the developmental trajectory of macrophage polarization in PDAC, we performed pseudotemporal ordering analysis. The trajectory reconstruction revealed two distinct differentiation pathways corresponding to M1 and M2 macrophage lineages (Figure 7I; cell fate 1: M1 macrophages; cell fate 2: M2 macrophages), corroborating previous findings 23. Transcriptomic profiling along the differentiation trajectory identified dynamic expression patterns of key regulatory genes involved in macrophage polarization, including *MKI67* and *UBE2C* (Figure 7J), providing mechanistic insights into the molecular regulation of macrophage phenotypic switching in the PDAC TME.

The scRNA-seq analysis revealed that elevated expression of hsa_circ_0058495 was associated with a significant expansion of M2 macrophage polarization within the TME of PDAC, accompanied by dynamic regulation of key genes (*MKI67* and *UBE2C*), thereby contributing to the establishment of an immunosuppressive microenvironment.

### EIF4A3 promotes the biosynthesis of hsa_circ_0058495

Recent research has revealed a crucial role for RNA-binding proteins (RBPs) in the biogenesis of exon-derived circRNAs. Specifically, RBPs have been shown to interact with the flanking sequences of circRNA-generating exons in pre-mRNA, facilitating the formation of circRNAs. We investigated this possibility using the Circinteractome and Starbase 3.0 databases, which predicted that EIF4A3 binds to RHBDD1 pre-mRNA in both intron3 and intron8 (Figure S7C). To investigate whether EIF4A3 regulated the biogenesis of hsa_circ_0058495, we transfected siRNAs targeting EIF4A3 (si-EIF4A3) and an over-expression plasmid (pLVX-EIF4A3) into PANC1 and BxPC3 cells. The RT-qPCR analysis demonstrated that EIF4A3 overexpression significantly increased hsa_circ_0058495 levels, while EIF4A3 knockdown suppressed hsa_circ_0058495 expression (Figure S7D). Subsequently, we amplified and quantified hsa_circ_0058495 from cDNA and gDNA with convergent or divergent primers in PANC1 cells with or without EIF4A3 knockdown. The results demonstrated that there was no significant change of PCR products from gDNA or cDNA using convergent primers after EIF4A3 knockdown. However, the PCR product was significantly reduced in cDNA with divergent primers following EIF4A3 knockdown. This confirmed that EIF4A3 depletion specifically downregulated mature hsa_circ_0058495 without altering genomic structure (Figure S7E). Then we detected the abundance of hsa_circ_0058495 in exosomes after EIF4A3 knockdown in PANC1 and BxPC3 cell lines. The results demonstrated that knockdown of EIF4A3 significantly downregulated hsa_circ_0058495 in exosomes from both cell lines (Figure S8A), consistent with its intracellular reduction in Figure S7D. Furthermore, an RNA-pull down assay confirmed that EIF4A3 directly interacted with intron3 and intron8 of RHBDD1 pre-mRNA (Figure S8B).

Based on the known role of hsa_circ_0058495 in promoting PDAC proliferation, we hypothesized that EIF4A3 contributed to PDAC progression by enhancing hsa_circ_0058495 biogenesis. To test this, we performed a CCK8 assay in PANC1 cells transfected with si-EIF4A3 or pLVX-EIF4A3. The results showed that EIF4A3 facilitated tumor cell growth (Figure S8C). To further confirm that EIF4A3 promoted PDAC by regulating hsa_circ_0058495, we performed an EdU assay in BxPC3 cells transfected with si-EIF4A3 in the presence or absence of pLC5-0058495. The results showed that hsa_circ_0058495 effectively rescued the proliferation of tumor cells that had been inhibited by EIF4A3 depletion (Figure S8D). Collectively, our findings indicated that EIF4A3 facilitated the cyclization of hsa_circ_0058495, which was also a carcinogenic component in PDAC.

## Discussion

Circular RNAs (circRNAs) have emerged as central regulators in the intricate network of cancer biology. Accumulating evidence underscores their critical roles in tumorigenesis and metastasis, influencing diverse cellular processes such as the maintenance of cancer stemness, angiogenesis, apoptosis and autophagy, as well as tumor invasion and metastatic dissemination 24-26. In the present study, we demonstrate that hsa_circ_0058495 is frequently upregulated in PDAC tissues, with EIF4A3 contributing to its biogenesis via back-splicing. Mechanistically, hsa_circ_0058495 stabilizes IGF2BP2 by attenuating autophagy-lysosome mediated degradation and concurrently protecting it from TRIM25-mediated ubiquitination. Acting as a pivotal m6A reader, IGF2BP2 subsequently enhances MEKK1 mRNA stability, thereby activating the ERK1/2 signaling cascade and promoting PDAC progression. Notably, hsa_circ_0058495 packaged within PDAC-derived exosomes is delivered to tumor-associated macrophages (TAMs), thus facilitating M2 macrophage polarization and further contributing to an immunosuppressive tumor microenvironment. Collectively, our findings reveal a previously unrecognized hsa_circ_0058495-IGF2BP2-MEKK1 axis, which drives PDAC progression and fosters a tumor-supportive microenvironment, highlighting hsa_circ_0058495 as a potential therapeutic target.

While confirming RHBDD1 gene upregulation in PDAC, our analysis reveals no significant correlation between RHBDD1 levels and patient prognosis. This suggests that bulk transcriptional activity at the RHBDD1 locus is neither a primary driver of aggressive PDAC behavior nor a robust prognostic indicator. However, there may exist some potential contributing factors led to the insignificance, such as the statistical power limitations inherent in the available TCGA-PAAD sample size and possible cohort heterogeneity, which needs future validation in larger independent cohorts. Critically, our findings establish hsa_circ_0058495, a circular RNA isoform derived from RHBDD1 pre-mRNA as a potent oncogenic effector. Hsa_circ_0058495 promotes key malignant phenotypes, including tumor cell proliferation and invasion, and functions as a strong independent prognostic biomarker predictive of poorer survival. This evidence confirms that the hsa_circ_0058495 plays its own unique biological role, independent from its parental gene *RHBDD1*, which may be owed to its inherent structural stability and unique mechanism via direct interaction with IGF2BP2 thus determining its specific biological impact and clinical relevance in PDAC pathogenesis.

Several RBPs have been found to regulate the biogenesis of circRNAs, such as QKI and EIF4A3 27, 28. EIF4A3 binds with the flanking regions of circRNA-forming exons, bringing them into close proximity to promote back splicing of circRNAs 29, 30. For instance, EIF4A3 binds to the upstream and downstream introns in SEPT9, ARHGAP29, TFRC, and SPIN1 RNA to induce the biogenesis of circRNAs in breast cancer, prostate cancer, and cervical cancer 14, 28, 31, 32.

The present study demonstrates that EIF4A3 binds to introns in the flanking regions of hsa_circ_0058495-forming exon 4-8 of RHBDD1 pre-mRNA to promote the generation of hsa_circ_0058495. Our study provides insights into the effects of EIF4A3-regulated circRNA on the development of PDAC.

Most studies have focused on the role of circRNAs as miRNA sponges or scaffolds. For example, hsa_circ_001783 promotes breast cancer by sponging miR-200c-3p 33, and circ_CEA acts as a scaffold to enhance the interaction of P53 and CDK1 to inhibit the apoptosis of gastric cancer cells 34. Our study reveals that hsa_circ_0058495 exerts its effect by stabilizing IGF2BP2 through inhibition of its degradation, rather than functioning as a ceRNA. On one hand, hsa_circ_0058495 suppresses the formation of autophagosomes and autolysosomes thus inhibiting the degradation of IGF2BP2 through the autophagy pathway. In addition, hsa_circ_0058495 binds with IGF2BP2 and affects the ubiquitin-ligase activity of TRIM25 for IGF2BP2 through steric hindrance. A previous study found that the K homologs of IGF2BP2 contribute to its function of binding to RNAs 35. Consistent with this, our data demonstrates that hsa_circ_0058495 is primarily bound to KH3-4 domains of IGF2BP2, which are the most frequent lysines ubiquitylated by TRIM25, rather than RRM1-2 or KH1-2. Therefore, we clarify that hsa_circ_0058495 protected IGF2BP2 from ubiquitination through steric hindrance, in which hsa_circ_0058495 binds with KH3-4 domains, thus blocking the combination of TRIM25 on IGF2BP2. A similar mechanism has also been reported in other studies. For example, circNEIL3 stabilizes IGF2BP3 by blocking and preventing HECTD4-mediated ubiquitination in glioma 36, and circ_0006646 prevents the interaction of NCL and TRIM21 thus inhibiting the TRIM21-mediated ubiquitinated-degradation of NCL in hepatocellular carcinoma 37.

N^6^-methyladenosine is the most prevalent post-transcriptional modification in eukaryotic cells, which is known to contribute to tumor progression by regulating mRNA stability and translation 38, 39. IGF2BP2, a crucial m6A-binding protein (reader), has been reported to bind to thousands of target RNAs and enhance mRNA stability by regulating the level of m6A modification 17. For instance, IGF2BP2 binds to the m6A-modified stop codon regions of TAB3 and promotes its stability 40. IGF2BP also stabilizes MYC, GPT2, and SLC1A5 mRNA to promote acute myeloid leukemia (AML) development 41. Our study identifies that MEKK1, a novel target of m6A modification, could be stabilized by IGF2BP2, thereby leading to the activation of ERK1/2 and promoting PDAC progression.

Exosomes play a pivotal role in facilitating tumor progression by serving as versatile nanocarriers that transport biological cargos including proteins, lipids, RNAs, and DNAs, between cancer cells and immune cells within the TME 42, 43. Through this intercellular crosstalk, exosomes modulate the immune response in the TME 44. Among these, exosomes-carried circRNAs have been widely reported to play a role in bladder cancer, prostate cancer, and endometrial cancer by regulating the differentiation or activation of CD8^+^ T cells, MDSC, and macrophages 5, 45, 46. However, very few exosomal-circRNAs have been reported in PDAC and its TME 47, 48. This study highlights the significant role of exosomal hsa_circ_0058495 in promoting PDAC proliferation, invasion, and migration. Notably, we found that hsa_circ_0058495 is more abundant in M2 macrophages than M1 macrophages in PDAC tissues. Furthermore, we reveal that hsa_circ_0058495 enhances M2 polarization through the IGF2BP2/MEKK1/p-ERK1/2 pathway.

We leverage the KPC mouse model which accurately mirrors human pancreatic cancer biology through targeted mutations in *Kras* and *Trp53* genes, using Cre-Lox technology. The KPC mouse is considered as an appropriate model for the study of pancreatic cancer. To investigate the tumor-promoting effects of exosomal hsa_circ_0058495 *in vivo*, we employed KPC mice as a spontaneous PDAC model and administered exosomes enriched with hsa_circ_0058495. We deemed the KPC mouse model suitable for evaluating the effects of exogenous hsa_circ_0058495 on tumor growth. Since the KPC mouse already expresses hsa_circ_0058495 endogenously within the tumorsphere, driven by the TME, we did not include a control group receiving exosomes with low levels of hsa_circ_0058495. Our results show that exosomal hsa_circ_0058495 significantly enhances the growth of PDAC *in vivo*.

In conclusion, our study reveals that hsa_circ_0058495 acts as an oncogene in PDAC, exerting its tumorigenic effects through two distinct mechanisms: firstly, by stabilizing IGF2BP2/MEKK1 and activating the p-ERK1/2 signaling pathway, and secondly, by promoting M2 macrophage polarization, thereby facilitating immune evasion. Our findings provide insights into the critical roles of hsa_circ_0058495 in PDAC progression and highlight its potential as a diagnostic and therapeutic target for PDAC.

## Methods

### Patients and tissue specimen collection

The PDAC tissues from patients who underwent surgery at The First Hospital of Jilin University between August 2019 and August 2022 were chosen. The postoperative pathological reports, independently confirmed by two experienced pathologists, verified the presence of PDAC. The tissues were immediately snap-frozen and stored in liquid nitrogen at -80 °C following dissection. The study protocol was approved by the ethics review board of The First Hospital of Jilin University. We have obtained written informed consent from all study participants. All of the procedures were performed in accordance with the Declaration of Helsinki and relevant policies in China.

### Cell lines and cell culture

The pancreatic cancer cell lines (PANC1, SW1990, BxPC3) and the normal pancreatic duct epithelial cell line (HPDE6C7) were acquired from ATCC. The human embryonic kidney 293T cell line was obtained from National Collection of Authenticated Cell Cultures (Shanghai, China). HPDE6C7 and BxPC3 cell lines were cultivated in 1640 medium (11875119, Gibco) supplemented with 10% fetal bovine serum (04-001-1ACS, BI) and 2% Penicillin-Streptomycin Solution. SW1990 cells were cultivated in DMEM-F12 medium (11320033, Thermo-Fisher) while PANC1 and 293T cells in DMEM medium (11965092, Gibco) supplemented with 10% fetal bovine serum and 2% Penicillin-Streptomycin Solution. The cells were incubated at 37 °C in an environment containing 5% carbon dioxide.

### Microarray analysis

The circRNA microarray analysis of exosomes was conducted by RIOBIO (Guangzhou). The raw sequencing dataset GSE79634 is publicly available on the GEO database (https://www.ncbi.nlm.nih.gov/geo). The differential expression of circRNAs was analyzed using the DEseq2 tool of R (version 4.2.1) based on the criteria of Log_2_^Fold Change^ (Log_2_^FC^) > 1.2 and p.adj ≤ 0.05. The microarray expression of circRNAs from 20 pairs of tumor and surrounding tissues was acquired from the GEO database, specifically from dataset GSE79634. The filter standard and analysis procedure were identical to those mentioned earlier.

### RNase R treatment

A total of 2 μg RNA was treated with 2 U/μg Rnase R (M0100S, NEB) at 37 °C for 15 minutes. Then the expression of hsa_circ_0058495 and RHBDD1 mRNA was tested by RT-qPCR.

### Actinomycin D assay

Cells were seeded onto six-well plates at a density of 5 × 10^5^ cells per well. After 24 hours, the cells were treated with 5 μg/mL of Actinomycin D (HY-17559, MCE) for different durations (0, 2, 4, 8, 12 and 24 hours). Subsequently, RNA was obtained via TRIzol reagent and RT-qPCR was conducted to assess the half-life of the target RNAs.

### Vector construction

The linear hsa_circ_0058495 was amplified via PCR from 293T cell lines. The primer sequences are presented in the Supplementary Table. Subsequently, it was introduced into the pLC5-ciR vector provided by Geenseed Biotech Co. The pLC5-0058495 was subsequently encapsulated into lentivirus for achieving stable transfection. The EIF4A3 overexpression vector was created by utilizing the pLVX-puro vector. The entire length of IGF2BP2 and its truncated versions, known as RRM1-2, KH1-2, and KH3-4, were inserted into the pLVX-puro vector. The linear hsa_circ_0058495 mutations were generated by PCR using Phusion High-Fidelity DNA Polymerase (M0530L, NEB) from the pLC5-0058495 vector and inserted into pLC5-ciR vector using the In-Fusion HD cloning kit (639649, Clontech). The si-0058495, si-IGF2BP2, and si-EIF4A3 were acquired from JTS bio (Wuhan).

### Transfection

A total of 1 × 10^5^ cells were placed in the six-well plate until attainment of 40% - 60% confluence. Transfection was then conducted using Lipo2000 (11668019, Thermo-Fisher) according to the manufacturer's instructions. The simultaneous introduction of two or more vectors into cells was carried out in a 1:1 (M/M) ratio. The transfection mixture was replaced after 6 hours, and the cells were thereafter grown with full media until the specified period.

### RT-PCR and RT-qPCR

The RNA extraction was performed using TRIzol (15596-018, Invitrogen) and the RNA concentration was determined using Nanodrop spectrophotometer. The reverse transcriptase was acquired from Takara (2680A) and the RT-qPCR procedure to generate cDNA was carried out using the BIO-RAD iCYCLER. The SYBR-Green dye was acquired from Takara (RR820A). Subsequently, the fluorescence quantification reaction in real time was observed using an MX-3000 system. The relative expression levels of the target RNAs were determined using the 2^-ΔΔ^CT^ method.

### Western blot analysis

The RIPA lysis buffer (89900) and the protease inhibitor cocktail (78429) were obtained from Thermo-Fisher. Each sampling hole was loaded with 40 µg of protein lysate, and separation was performed using a 10% SDS-PAGE. Subsequently, the protein was deposited onto a PVDF membrane and incubated overnight with the following primary antibodies at 4 ℃: anti-IGF2BP2 antibody (14672, CST), anti-EIF4A3 antibody (bs-14548R, Bioss), MAPK Family antibody sampler kit (9926, CST), phospho-MAPK family antibody sampler kit (9910, CST), anti-ubiquitin antibody (ab134953, Abcam), anti-K48 antibody (4289, CST), anti-Flag antibody (66008-4-Ig, Proteintech), anti-HA antibody (51064-2-AP, Proteintech), anti-LC3A/B antibody (12741, CST), anti-SQSTM1 antibody (sc-26675, Santa Cruz), anti-TRIM25 antibody (67314-1-Ig, Proteintech), anti-Arginase-1 antibody (16001-1-AP, Proteintech), anti-iNOS antibody (18985-1-AP, Proteintech), anti-CD206 antibody (81525-1-RR, Proteintech), anti-CD86 antibody (13395-1-AP, Proteintech), anti-α-Tubulin antibody (sc-8035, Santa Cruz), anti-β-actin antibody (1030300012, lifespaceo). This was followed by hybridization with an HRP-conjugated secondary antibody at room temperature for 40 minutes. The immunoreactive signals were detected using a chemiluminescence system (Bio-Rad, United States).

### Wound healing assay

Each well of the six-well plates was seeded with 5 × 10^5^ cells. After the cell confluence had reached 80% - 90%, a scratch was created using a 200 μL pipette tip by drawing a straight line. The isolated cells were rinsed thrice with PBS. Subsequently, the wound healing process in the scratched region was monitored and photographs were obtained at intervals of 0, 12, 24, and 48 hours. The size of the fusion area was determined using Image J software.

### Cell viability assay

Cell viability assay was conducted using the cell counting kit-8 (C0038, Beyotime). Each well of the 96-well plates was seeded with 1 × 10^5^ cells and 10 μL CCK-8 solution was added at 0, 24, 48, and 72 h. After 4 hours incubation, the absorbance readings were taken at 450 nm with a microplate reader (BioRad, USA).

### EdU assay

Fluorescent EdU detection was employed by Cell-Light EdU Apollo 488 *In vitro* Kit (C10310-3, Ribobio). 2 × 10^5^ cells was seeded into each well of the 48-well plates. Then EdU reagent was added and incubated for 2 hours and then fixed using 4% paraformaldehyde (P0099, Beyotime). Samples were examined with a fluorescence microscope (Leica DMI6000B) following the application of the suitable dye.

### Single-Cell RNA Sequencing and Analysis

The scRNA-seq experiments were outsourced to Singleron Biotechnologies (Nanjing, China), utilizing their proprietary GEXSCOPE® platform for high-resolution single-cell profiling. Tumor tissues were enzymatically dissociated into single-cell suspensions using a standardized protocol. Viable cells were isolated via fluorescence-activated cell sorting (FACS) and processed for scRNA-seq library preparation using the GEXSCOPE® Single-Cell RNA Sequencing Kit. Sequencing was carried out on the Illumina NovaSeq 6000 platform, generating paired-end reads with an average depth of 50,000 reads per cell to ensure robust transcriptomic coverage. Raw sequencing data were processed using Singleron's SCOPE pipline, which included demultiplexing, barcode assignment, and unique molecular identifier (UMI) counting. Cells with fewer than 200 detected genes or more than 6,000 genes were excluded to remove low-quality cells and potential doublets. Additionally, cells with mitochondrial gene content exceeding 20% were filtered out to eliminate stressed or apoptotic cells.

### RNA pull-down assay

The RNA probe was labeled with desulphurization biotin at the 3' end using the Pierce RNA 3' End Desthiobiotinylation Kit RNA (20163, Thermo-Fisher). The biotin-labeled hsa_circ_0058495 probe was acquired from Gema (China). The RNA-pull down experiment was conducted using the Pierce Magnetic RNA-Protein Pull-Down Kit (20164, Thermo-Fisher) according to the manufacturer's instructions. Proteins interacting with RNA were examined using mass spectrometry and Western blot analysis.

### RNA immunoprecipitation

RNA Immunoprecipitation Kit (P0101, Geenseed, China) was used for RNA immunoprecipitation. 1 × 10^7^ cells were lysed using RIPA buffer containing 1% protease inhibitor cocktail. The anti-Flag and IgG antibodies were incubated with protein A/G beads at 4 ℃ for 4 hours. Subsequently, the beads were incubated overnight with cell lysate at 4 ℃. The beads were rinsed several times with RIP wash buffer and then suspended in Proteinase K Buffer. Subsequently, the RNA was isolated and purified using chloroform and anhydrous ethanol. Then cDNA was obtained using reverse transcription. Finally, hsa_circ_0058495 expression was determined using RT-qPCR.

### Co-immunoprecipitation

Co-immunoprecipitation was performed using the Pierce Co-immunoprecipitation Kit (88804, Thermo-Fisher) according to the manufacturer's instructions. The cells were lysed using Pierce IP Lysis Buffer (87788, Thermo-Fisher) and subsequently incubated with protein A/G beads that were conjugated overnight with anti-IGF2BP2 and IgG antibodies at 4 °C. Following several washes, the immunoprecipitated proteins were eluted and analyzed using mass spectrometry and Western blot assay.

### *In vivo* tumorigenicity assay

The animal study was executed following the approval of the ethics committee of the first hospital of Jilin University. The Kras^G12D/+^ Trp53R^172H/+^ Pdx-1-Cre (KPC) mice were gained from Cyagen Biosciences. At 8 weeks of age, KPC mice were randomly divided into two groups and then injected via tail veins with 60 μL of PBS containing exosomes with or without hsa_circ_0058495. The exosomes were extracted from BxPC3 cell lines overexpressing hsa_circ_0058495 or a blank plasmid. The exosomes were injected once a week and the tumors were scanned using PET-CT 6 weeks later. Then the mice were euthanized, and the tumors were resected and measured. The tumor size was calculated using formula volume (mm^3^) = width^2^ × length/2. After that, flash-frozen and formalin-fixed, paraffin-embedded (FFPE) samples were taken for histological analyses.

### Orthotopic mouse model

All animal experimental protocols were performed in accordance with the National Institutes of Health Guidelines for the Care and Use of Experimental Animals. C57BL/6 and NOD-SCID mice were injected with Pan02 tumor cell suspensions (5 × 10⁴ to 1 × 10⁵ cells/injection in Matrigel, 354230; Corning) directly into the pancreas, followed by closure of the peritoneum with 5-0 Vicryl sutures and the skin with surgical staples. Tumors were allowed to grow for 2 weeks prior to exosomes treatment.

### PDX mouse model

To generate patient-derived xenografts (PDXs), surgical specimens from primary tumors of the patient were immediately implanted in mice. The tumor was resected from a 71-year-old woman, and the pathological diagnosis revealed a 3.2 × 3 × 2.5 cm pancreatic ductal adenocarcinoma, moderately differentiated, staged as T2N2M0. Fragments of 8 mm3 were implanted into the lower flank of 6-week-old female Balb/c nude mice (SPF biotechnology, China). Animals were housed in laminar flow cabinets under a 12-hour light/dark cycle with unrestricted access to food and water. Established tumors were serially passaged to maintain the models.

### Flow cytometry

The cells were suspended to a density of 1×10^6^ cells/ml, and 5 μL of F4/80, CD86 and CD206 staining solution were added to 300 μL of the cell suspension. After an incubation for 10-15 min at room temperature in the dark, stained cells were assayed and quantified using a FAC Sort Flow Cytometer (BD, San Jose, CA, USA). Each experiment was performed in triplicate and repeated at least twice.

### ELISA

Conditioned media or cellular protein lysates were quantified, and equal protein amounts were loaded per well of a 96-well plate with three technical replicates per biological replicate. ELISA kits specific for human IL-1β, TNF-α, TGF-β, and IL-10 (Invitrogen, A35574, A42898, BMS249-4, and BMS215INST) were used according to manufacturer protocol to quantify cytokines levels. Results were measured by the absorbance of each well using a BioRad microplate reader.

### Statistical analysis

The results were presented as mean ± standard deviation (SD) and between-group differences were assessed for statistical significance using variance analysis followed by unpaired, two-tailed student's test. For comparison among multiple groups, the ANOVA test was used. The Pearson chi-square test was used for testing the differences between categorical variables. Fisher's exact test was used when the number of variables was lower than 5. The statistical analysis was conducted using Prism software (GraphPad Software 9.0) and SPSS (IBM SPSS Statistics 24.0). In general, P value < 0.05 was considered significant and was indicated as follows: * P < 0.05, **P < 0.01, ***P < 0.001, ****P < 0.0001, ns: no significance.

## Supplementary Material

Supplementary figures and tables.

## Figures and Tables

**Table 1:** Statistical analysis of clinical characteristics

Clinicopathological	Cases	hsa_circ_0058495 level		χ^2^	p-value
Parameters	70	High (35)	Low (35)		
**Age^a^**				1.596	0.299
<60	22	15	7		
≥60	48	25	23		
**Gender^a^**				0.123	0.811
Female	36	20	16		
Male	34	18	16		
**Tumor location^a^**				1.222	0.315
Head	47	22	25		
Body and tail	23	14	9		
**Vascular invasion^a^**				7.202	0.013*
Yes	56	38	18		
No	14	4	10		
**Nerve invasion^a^**				6.605	0.026*
Yes	60	43	17		
No	10	3	7		
**Degree of differentiation^b^**				0.932	0.730
Highly	3	1	2		
Moderately	15	9	6		
Poorly	52	31	21		
**T stage^b^**				14.745	0.001**
T1	6	2	4		
T2	28	11	17		
T3	17	12	5		
T4	19	17	2		
**Lymph node metastasis^b^**				11.350	0.004**
N0	23	8	15		
N1	29	23	6		
N2	18	13	5		
**TNM stage^a^**				6.564	0.038*
I	20	6	14		
II	36	21	15		
III	14	10	4		

a, Pearson chi-square test; b, Fisher's exact test. * *P* < 0.05, ** *P* < 0.01 TNM stage is classified according to the 8th edition of the American Joint Committee on Cancer (AJCC).

## Abbreviations

ActD: actinomycin D
AML: acute myeloid leukemia
CHX: cycloheximide
CI: confidence interval
circRNAs: circular RNAs
CRC: colorectal carcinoma
CRD: coding region instability determinant
DAA: 3-Denitroadenosine
ECs: endothelial cells
FACS: fluorescence-activated cell sorting
FISH: fluorescence *in situ* hybridization
GEO: gene Expression Omnibus
HR: hazard ratio
IGF2BP2: insulin-like growth factor 2 mRNA-binding protein 2
IF: immunofluorescence
IHC: immunohistochemistry
IP: immunoprecipitation
KH: K homology
mIf: multiplex immunofluorescence
MPs: mononuclear phagocytes
MS: mass spectrometry
NC: negative control
OS: overall survival
PDAC: pancreatic ductal adenocarcinoma
PDX: patient-derived xenograft
PanSCs: pancreatic stellate cells
PTMs: post-translational modifications
Rapa: rapamycin
RIP: RNA immunoprecipitation
RRM: RNA recognition motifs
SUV: standard uptake value
TAMs: tumor-associated macrophages
TEM: transmission electron microscopy
TME: tumor microenvironment
TNBC: triple-negative breast cancer
UTR: untranslated region
3-MA: 3-methyladenine
