# Supplementary material for: Hsa_circ_0058495-mediated IGF2BP2 ubiquitination and m6A modification of MEKK1 promote the progression of PDAC
Source: Theranostics. 2025 Sep 12;15(18):9922–43. doi: 10.7150/thno.117202 (PMC12486403; doi:10.7150/thno.117202)
Supplement: Supplementary file 1 — Supplementary figures and tables. [file thnov15p9922s1.zip › supplementary files/Supporting Information1.pdf]

Supporting Information

Figure S1-S8

Figure S1

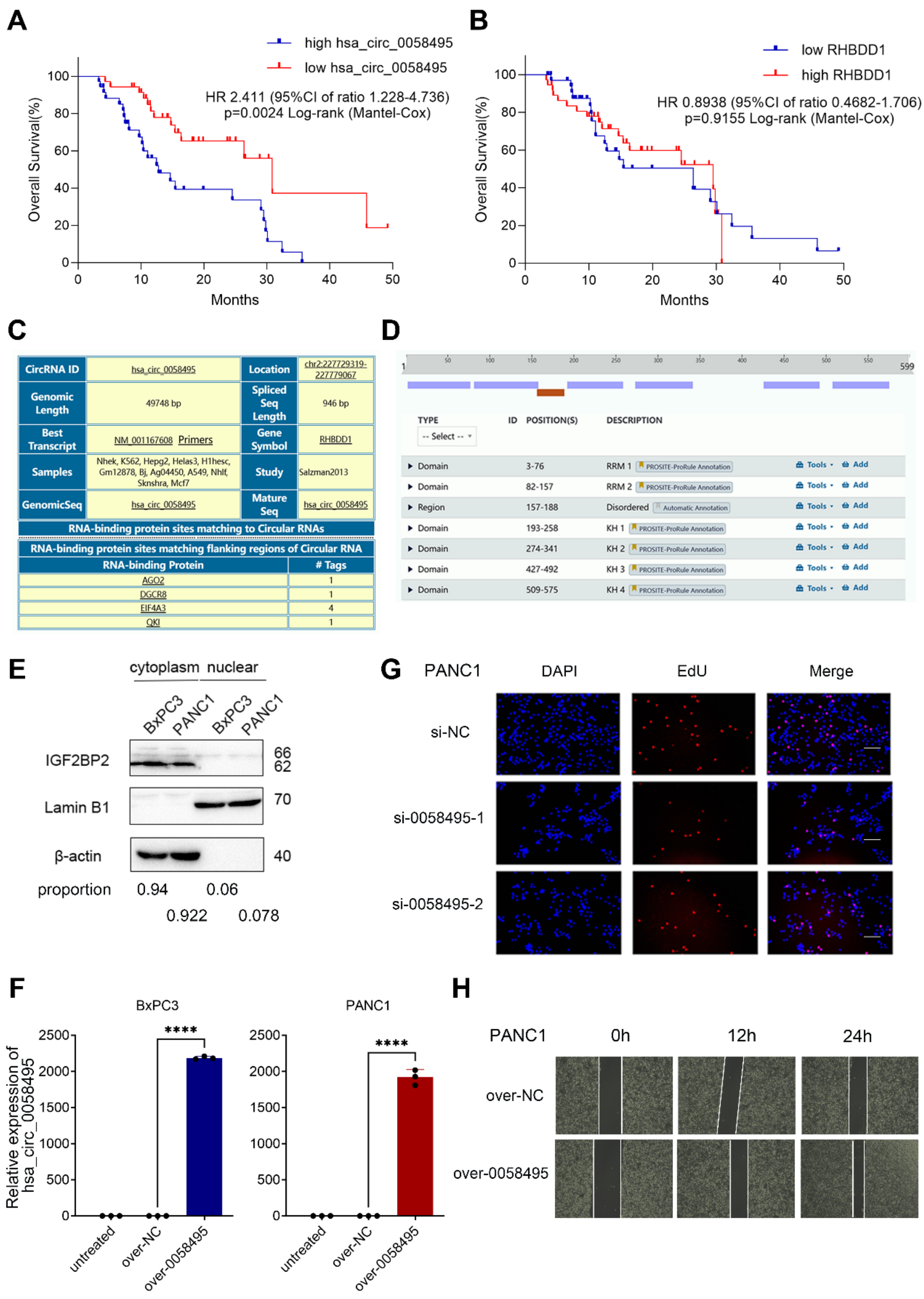

Figure S1. (A-B) Kaplan-Meier survival analysis showing the correlation between hsa_circ_0058495 (A)， RHBDD1 mRNA (B) levels and overall survival in our PDAC patient cohort. (C) The information of hsa_circ_0058495 was shown by circinteractome. (D) The analysis of IGF2BP2 domains was shown by uniport. (E) Cytoplasmic and nuclear protein fractionation and Western blot analysis was performed to analyze the subcellular localization of IGF2BP2 proteins in BxPC3 and PANC1 cells. (F) BxPC3 and PANC1 cells were transfected with plasmid expressing hsa_circ_0058495 for 24 hours. The level of hsa_circ_0058495 was determined by RT-qPCR. (G) PANC1 cells were transfected with siRNA against hsa_circ_0058495 si-NC for 24 hours. EdU assay was performed to assess the proliferation ability of cells. Proliferated cells (red) and nuclei (blue) are shown. Counts of total cells and proliferated cells are shown in the column. Scale bar, 40 μm. (H) PANC1 cells were transfected with over-0058495 or over-NC for 24 hours and wound healing assay was used to determine invasion ability. The cell invasion rate was calculated and shown in the column. Scale bar, 40 μm. ns, no significant; $*P < 0.05$; $**P < 0.01$; $***P < 0.001$; $****P < 0.\ 0001$.

Figure S2

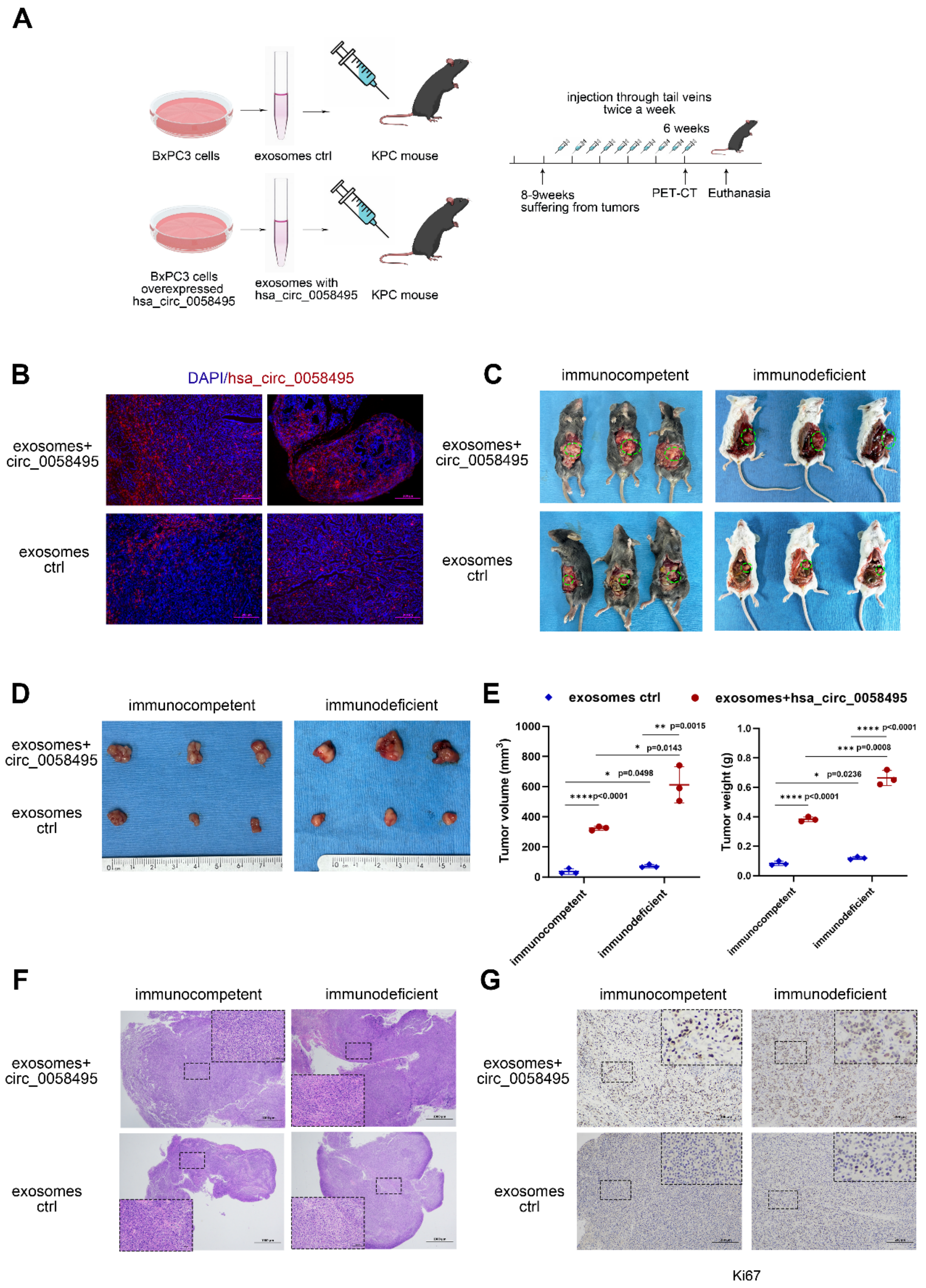

Figure S2. (A) Representation of the treatment administered to KPC mice. (B) Fluorescence in situ hybridization (FISH) assays showing the abundance of

hsa_circ_0058495 in PDAC tissues from KPC mice that treated with exosomes over-expressed hsa_circ_0058495 or not. Hsa_circ_0058495 (red) and nuclei (blue) are shown. Scale bar, 200 μm. (C) Representative photographs of C57BL/6 and NOD-SCID mice showing the orthotopic PDAC tumors in pancreas. (D) Displayed of harvested pancreatic tumor from orthotopic mice models, n = 3 mice per group. (E) The volume and weight of tumor lesions in each mouse were measured, n = 3 per group. (F) Representative images for H&E staining of mouse PDAC tissues. Scale bar, 1000 μm. (G) Representative images of Ki67 IHC analysis of mice PDAC lesions. Scale bar, 200 μm. ns, no significant; **P* < 0.05; ***P* < 0.01; ****P* < 0.001; *****P* < 0. 0001.

Figure S3

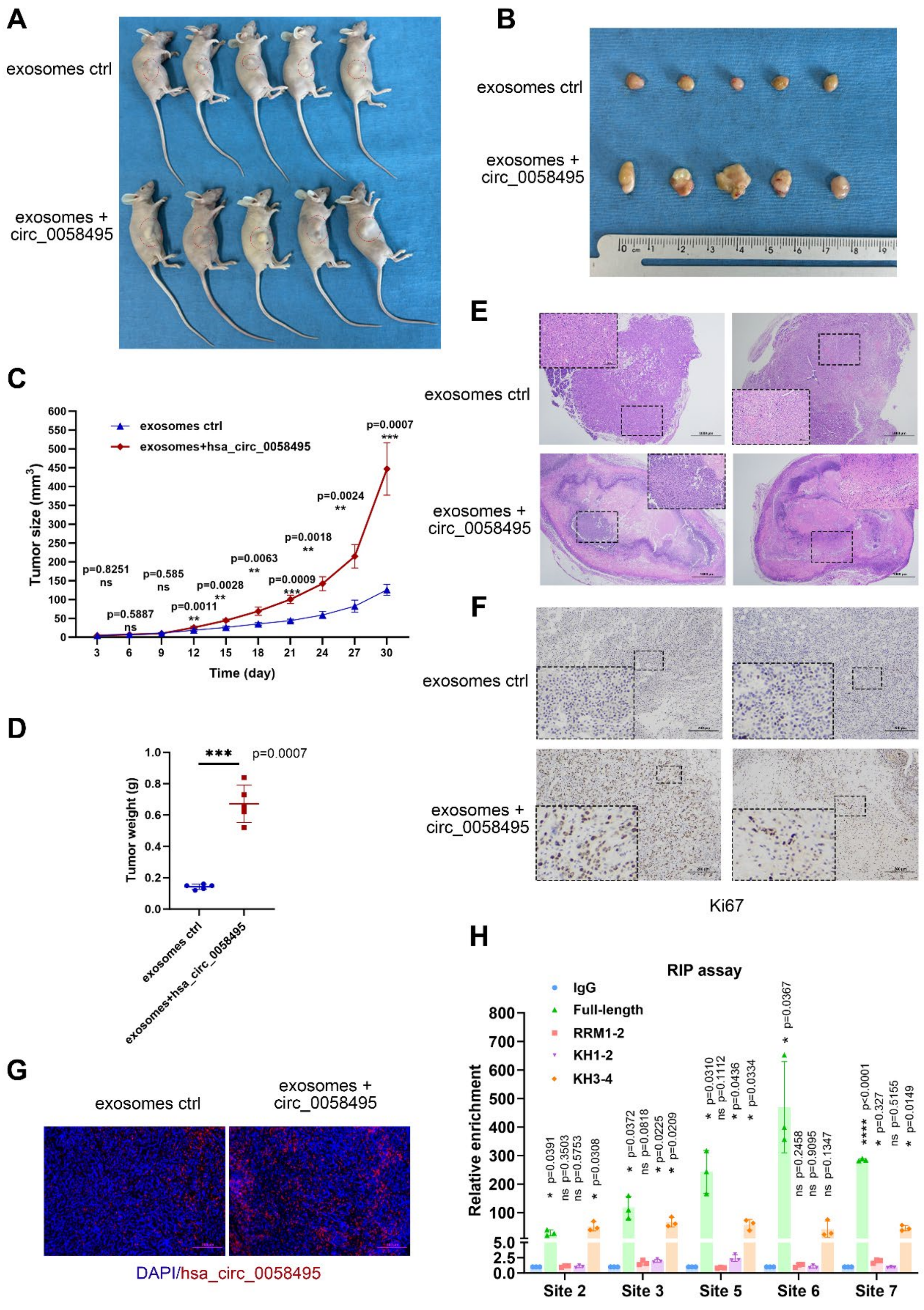

Figure S3. (A) Representative photographs of Balb/c nude mice with patient-derived PDAC tissues implanted subcutaneously in the flank. (B) Displayed of harvested

pancreatic tumor from PDX models, n = 5 mice per group. (C) The size of tumors in each mouse were measured each 3 days, n = 5 per group. (D) The weight of tumor lesions in each mouse were measured, n = 5 per group. (E) Representative images for H&E staining of mouse PDAC tissues. Scale bar, 1000 μm. (F) Representative images of Ki67 IHC analysis of mice PDAC lesions. Scale bar, 200 μm. (G) FISH assays showing the abundance of hsa_circ_0058495 in tumor tissues from PDX mice. (H) RNA-immunoprecipitation and RT-qPCR were performed to detect the combination of IGF2BP2 full length, RRM1-2, KH1-2 and KH3-4 domains with the m6A sites of MEKK1 mRNA. ns, no significant; **P* < 0.05; ***P* < 0.01; ****P* < 0.001; *****P* < 0. 0001.

Figure S4

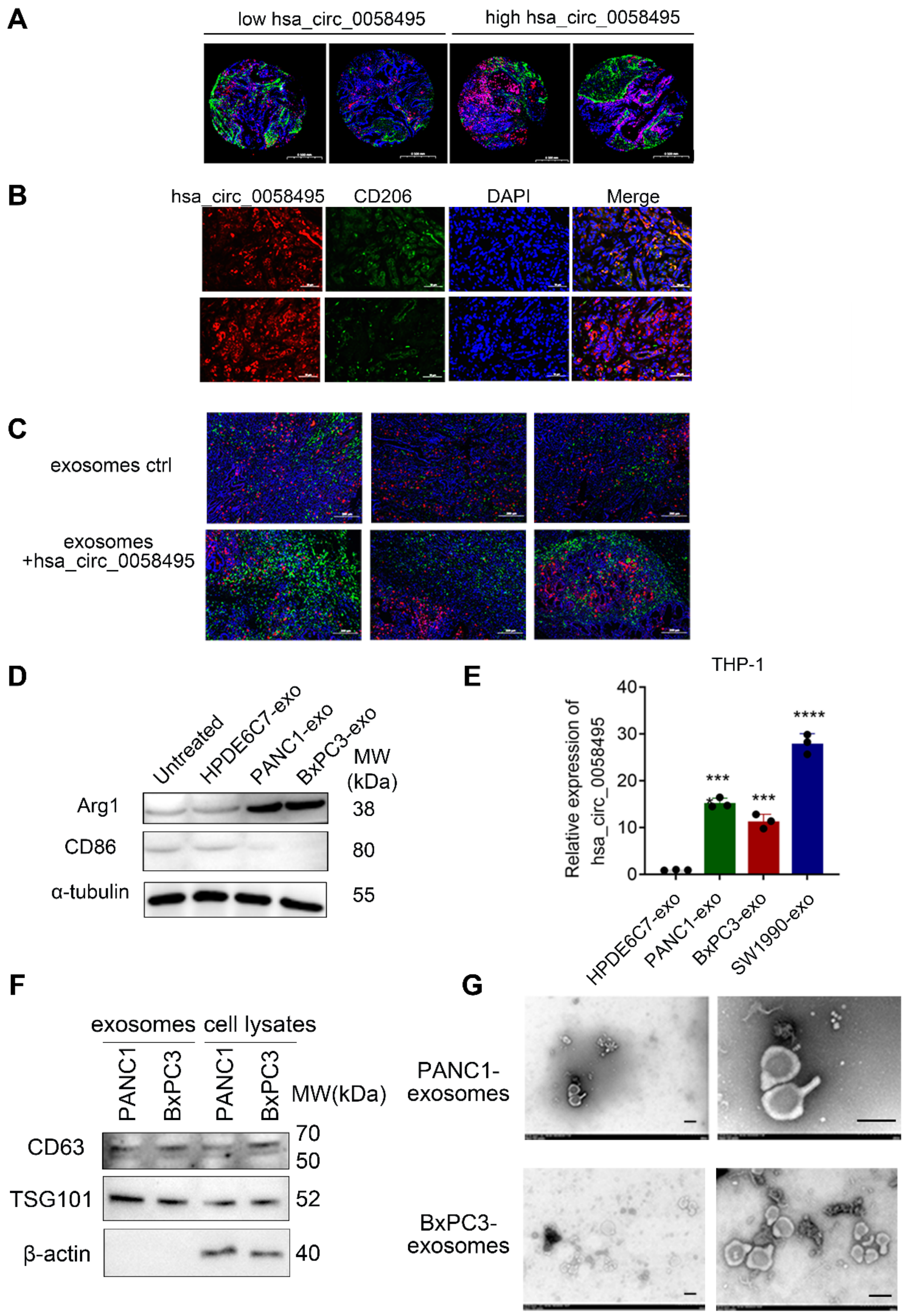

Figure S4. (A) Multiplex immunofluorescence (mIF) showed the infiltration of M1, M2 macrophages and cancer associated fibroblast (CAF) in PDAC tissues with high or

low hsa_circ_0058495 expression level. Scale bar, 500 μm. M1 macrophage (iNOS: red), CAF (α-SMA: green), and M2 macrophage (CD206: pink). (B) IF and FISH assays showed the abundance of hsa_circ_0058495 (red), CD206 (green) in PDAC tissues with DAPI staining shown as blue. Scale bar, 100 μm. (C) IF showed the distribution of M1 and M2 macrophage in tumor tissues of KPC mice injected exosomes overexpressed hsa_circ_0058495 or not through tail veins. Scale bar, 200 μm. M1 macrophage (iNOS: red), and M2 macrophage (CD206: green). (D) THP-1 cells were co-cultured with exosomes from HPDE6C7, PANC1, and BxPC3 cells for 48 hours. Arg1 and CD86 protein levels were determined by immunoblotting using α-tubulin as loading control. (E) THP-1 cells were co-cultured with exosomes from HPDE6C7, PANC1, and BxPC3 cells for 48 hours. The level of hsa_circ_0058495 in THP-1 cells was determined by RT-qPCR. (F) The markers of exosomes, CD63 and TSG101 protein levels were determined by immunoblotting using β-actin as control. (G) The morphology of exosomes from PANC1 and BxPC3 cells shown by transmission electron microscopy. Scale bar, 100 μm. ns, no significant; *$P < 0.05$; **$P < 0.01$; ***$P < 0.001$; ****$P < 0.\ 0001$.

Figure S5

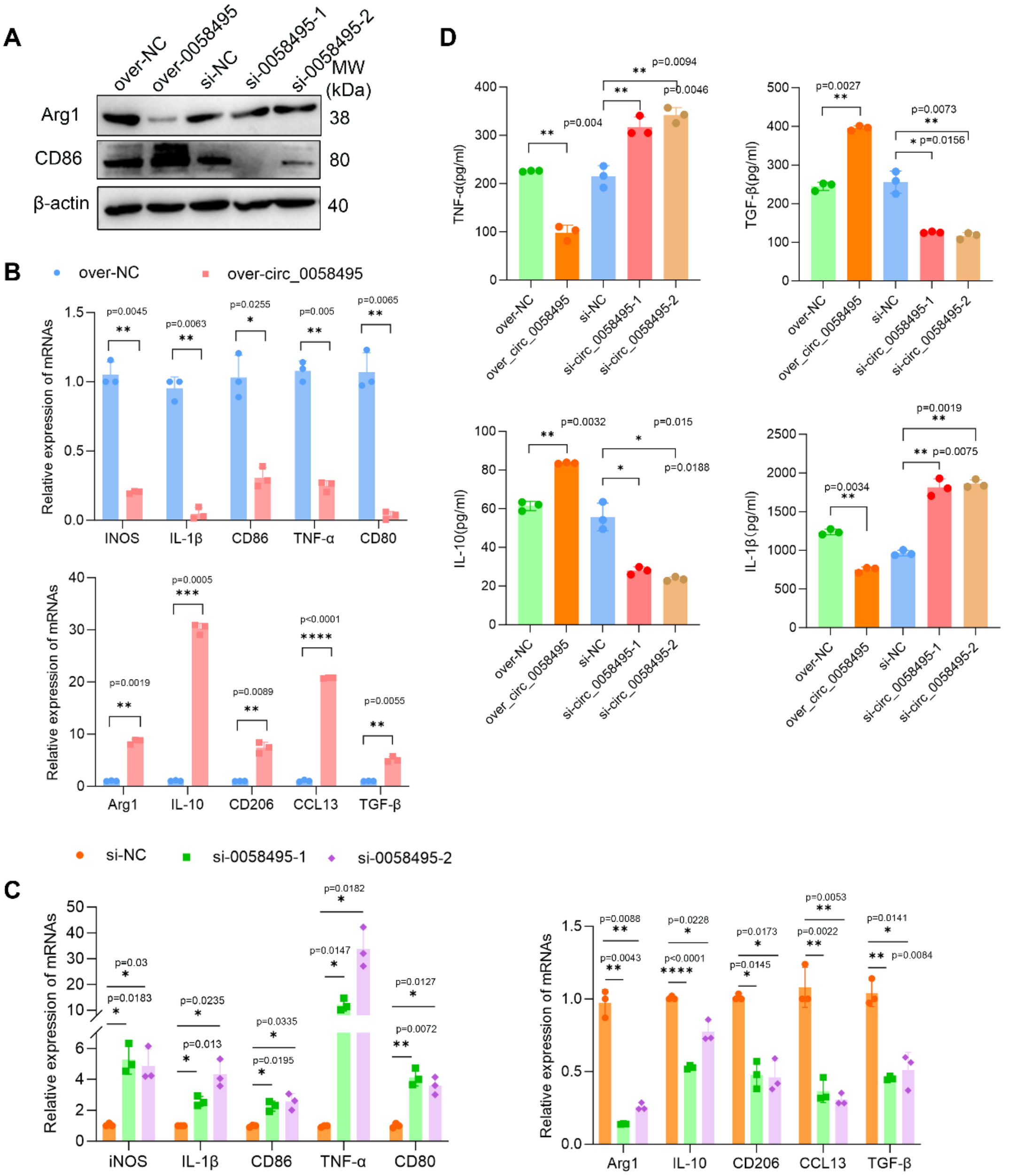

Figure S5. (A) THP-1 cells were transfected with over-NC or over-0058495, si-NC or si-0058495 for 48 hours. Arg1 and CD86 protein levels were determined by immunoblotting with β-actin as loading control. (B-C) THP-1 cells were transfected with over-NC or over-0058495 (B), si-NC or si-0058495 (C) for 24 hours. The levels of M1 markers (iNOS, IL-1β，CD86, TNF-α and CD80) and M2 markers (Arg1, IL-10, CD206, CCL13 and TGF-β) were detected by RT-PCR. (D) THP-1 cells were

transfected with over-NC or over-0058495, si-NC or si-0058495 for 48 hours. The levels of M1 markers (IL-1β, TNF-α) and M2 markers (IL-10, TGF-β) in the supernatant were detected by ELISA. THP-1 cells were transfected with si-NC or si-0058495 for 24 hours. ns, no significant; *$P < 0.05$; **$P < 0.01$; ***$P < 0.001$; ****$P < 0.\ 0001$.

Figure S6

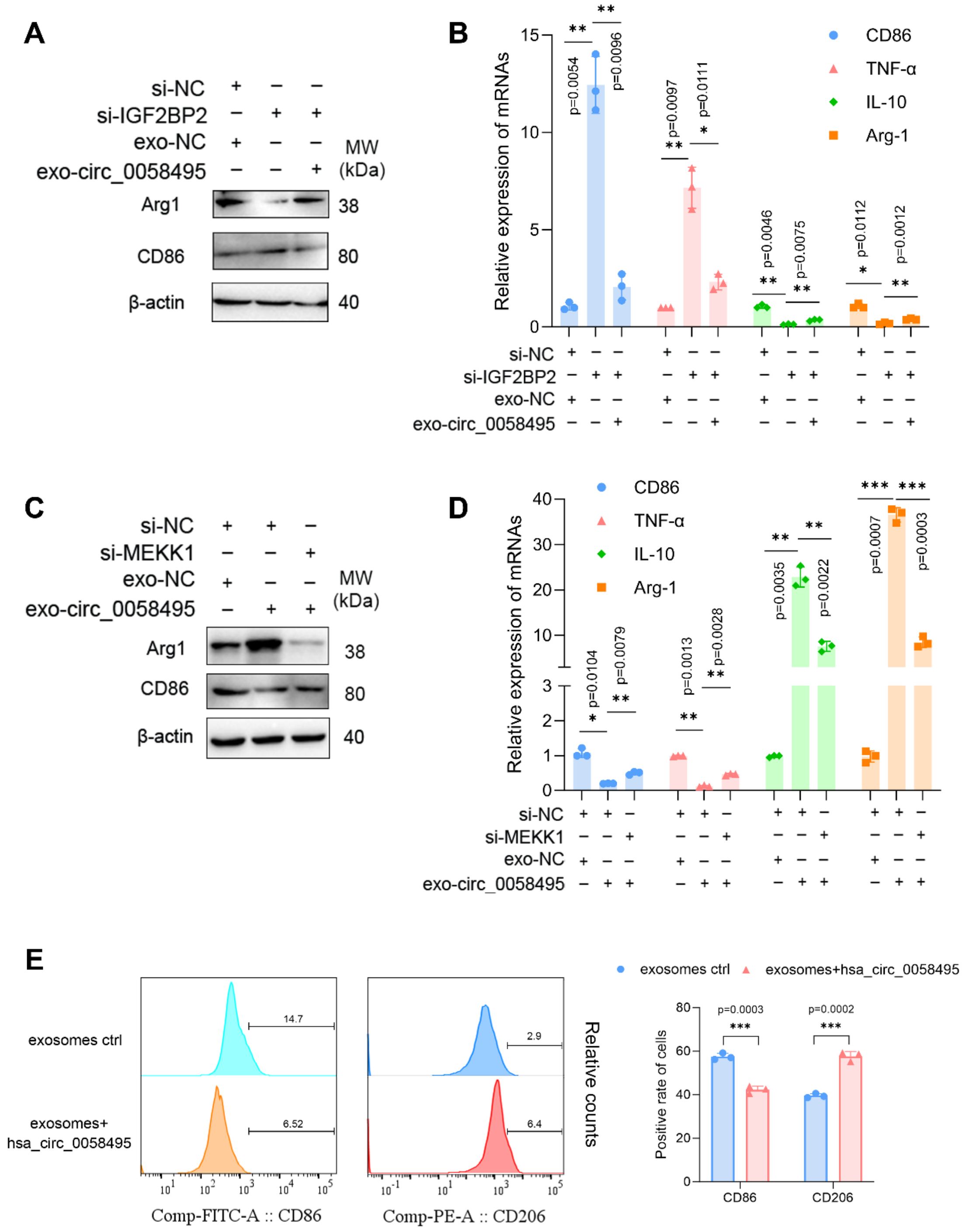

Figure S6. (A-B) THP-1 cells were transfected with si-NC or si-IGF2BP2 for 24 hours, then treated by exosomes derived from hsa_circ_0058495-overexpressed BxPC3 cells

for 48 hours. Arg1 and CD86 protein levels were determined by immunoblotting with β-actin as loading control (A). The level of CD86, TNF-α, IL-10, Arg-1 were detected by RT-qPCR (B). (C-D) THP-1 cells were transfected with si-NC or si-MEKK1 for 24 hours, then treated by exosomes derived from hsa_circ_0058495-overexpressed BxPC3 cells for 48 hours. Arg1 and CD86 protein levels were determined by immunoblotting with β-actin as loading control (C). The level of CD86, TNF-α, IL-10, Arg-1 were detected by RT-qPCR (D). (E) Flow cytometry assays showing the proportion of M2 ($CD206^{+}$) and M1 ($CD86^{+}$) in PDAC tissues form PDX mice models. ns, no significant; *$P < 0.05$; **$P < 0.01$; ***$P < 0.001$; ****$P < 0.0001$.

Figure S7

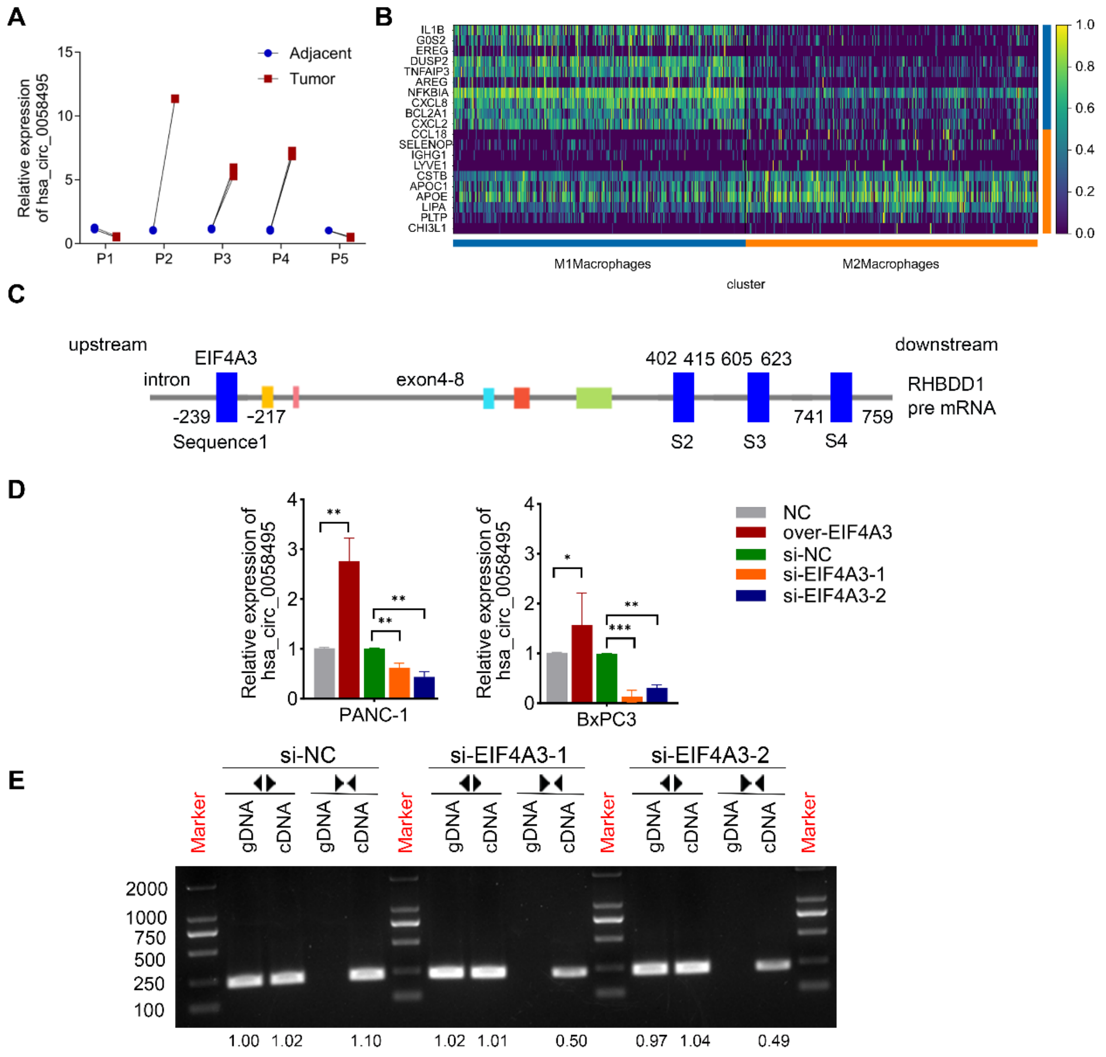

Figure S7. (A) RT-qPCR showed the level of hsa_circ_0058495 of five PDAC tumor tissues compared to adjacent tissues. (B) Differential expression heatmap of cluster-specific markers of M1 and M2 macrophages. (C) Schematic illustration showing the binding sites of EIF4A3 on the flanking regions of pre-RHBDD1 mRNA. (D) BxPC3 and PANC1 cells were transfected with siRNA against EIF4A3 or plasmid expressing EIF4A3 for 24 hours. (E) Agarose gel electrophoresis analysis of PCR product amplified by divergent and convergent primers of hsa_circ_0058495 from gDNA and

cDNA in PANC1 cells knockdown EIF4A3 or not. ns, no significant; *$P < 0.05$; **$P < 0.01$; ***$P < 0.001$; ****$P < 0.0001$.

Figure S8

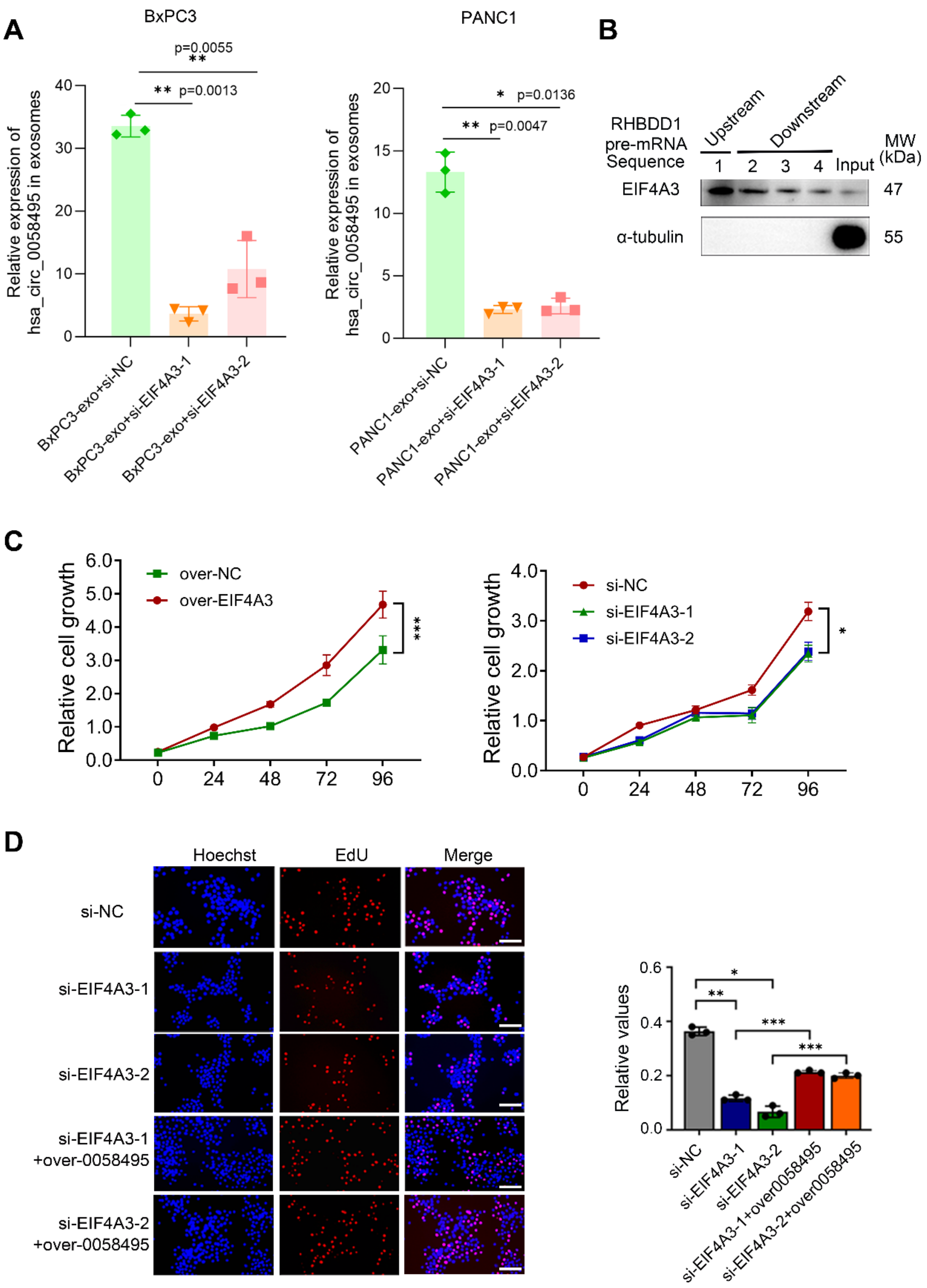

Figure S8. (A) RT-qPCR showing the abundance of hsa_circ_0058495 in exosomes in PANC1 and BxPC3 cells knockdown EIF4A3 or not. (B) RNA-pull down assay was

performed using biotin-labeled RNA probes for potential binding sequences of EIF4A3. The level of EIF4A3 pulled down was determined by immunoblotting. α-tubulin was probed as loading controls of input. (C) BxPC3 and PANC1 cells were transfected with siRNA against EIF4A3 or plasmid expressing EIF4A3 for 24 hours. CCK8 assay was used to determine cell growth rate. (D) BxPC3 and PANC1 cells were transfected with siRNA against EIF4A3 in the presence or absence of hsa_circ_0058495 for 24 hours. EdU assay was performed to detect the proliferation ability of cells. The proliferated cells (red) and nuclei (blue) are shown. Counts of total cells and proliferated cells are shown in the column. Scale bar, 40 μm. ns, no significant; *$P < 0.05$; **$P < 0.01$; ***$P < 0.001$; ****$P < 0.0001$.

qPCR Primers	
has_circ_0058495 divergent Primer Forward	ATGACACGTACACAGCAGGAC
has_circ_0058495 divergent Primer Reverse	CTCAGGTGGTCAGTTTCAGGT
has_circ_0058495 Convergent Primer Forward	ATATACAGACGGCTGAACCTCGGT
has_circ_0058495 Convergent Primer Reverse	GGCCAGATCACTATGAAGAAGCA
GAPDH F	GACAGTCAGCCGCATCTTCT
GAPDH R	GCGCCCAATACGACCAAATC
iNOS(H) F	GCTCTACACCTCCAATGTGACC
iNOS(H) R	CTGCCGAGATTTGAGCCTCATG
TNF-α(H) F	CTCTTCTGCCTGCTGCACTTTG
TNF-α(H) R	ATGGGCTACAGGCTTGTCACTC
CD86(H) F	CCATCAGCTTGTCTGTTTCATTCC
CD86(H) R	GCTGTAATCCAAGGAATGTGGTC
CD80(H) F	CTCTTGGTGCTGGCTGGTCTTT
CD80(H) R	GCCAGTAGATGCGAGTTTGTGC
CCL13(H) F	GATCTCCTTGCAGAGGCTGAAG
CCL13(H) R	TCTGGACCCACTTCTCCTTTGG
TGF-β(H) F	TACCTGAACCCGTGTTGCTCTC
TGF-β(H) R	GTTGCTGAGGTATCGCCAGGAA
CD206(H) F	AGCCAACACCAGCTCCTCAAGA

CD206(H) R	CAAAACGCTCGCGCATTGTCCA
CD163(H) F	CCAGAAGGAACTTGTAGCCACAG
CD163(H) R	CAGGCACCAAGCGTTTTGAGCT
EIF4A3-F	GGCACAGGAAAAACAGCCACCT
EIF4A3-R	TGTAGTCACCGAGAGCAAGCAG
IGF2BP2-F	GTTGGTGCCATCATCGGAAAGG
IGF2BP2-R	TGGATGGTGACAGGCTTCTCTG
MEKK1-F	CCAGACCAGTATCTCAGGAGATG
MEKK1-R	CCGCTAAACTGTGGCAAGGAGT
RHBDD1-F	GTTGGTTACCCAGGACGGCAAT
RHBDD1-R	CTTCACTCAGTCCTGCTGTGTAC

Primers for constructing over-expression vectors		
IGF2BP2: RRM1-2 F	CCGGAATTCATGATGAACAAGCTTTACATC	pcDNA 3.1+
IGF2BP2: RRM1-2 R	CCGCTCGAGTCAGGCGTAGTCAGGCACGTCGTATGGG TAATCCGGGATGTAGGAAAT	pcDNA 3.1+
IGF2BP2: KH1-2 F	CCGGAATTCATGCCGCTGCGGATCCTGGTC	pcDNA 3.1+
IGF2BP2: KH1-2 R	CCGCTCGAGTCAGGCGTAGTCAGGCACGTCGTATGGG TAATCATTTTCAAAGGCCTC	pcDNA 3.1+
IGF2BP2: KH3-4 F	CCGGAATTCATGGAGCAGGAGATTGTGAAT	pcDNA 3.1+
IGF2BP2: KH3-4 R	CCGCTCGAGTCAGGCGTAGTCAGGCACGTCGTATGGG TACTCCTGCTGCTTCACCT	pcDNA 3.1+

Over IGF2BP2 F	CCGGAATTCATGATGAACAAGCTTTACATC	pcDNA 3.1+
Over IGF2BP2 R	CCGCTCGAGTCAGGCGTAGTCAGGCACGTCGTATGGG TACTTGCTGCGCTGTGAGGC	pcDNA 3.1+
Over EIF4A3 F	CGCGGATCCATGGCGACCACGGCCACGATG	pcDNA 3.1+
Over EIF4A3 R	CCGCTCGAGTCAGGCGTAGTCAGGCACGTCGTATGGG TAGATAAGATCAGCAAC	pcDNA 3.1+

**siRNAs**	
hsa_circ_0058495-1-F	GACCGAGGUUCAGCCGUCUTT
hsa_circ_0058495-1-R	AGACGGCUGAACCUCGGUCTT
hsa_circ_0058495-2-F	GGGACCGAGGUUCAGCCGUTT
hsa_circ_0058495-2-R	ACGGCUGAACCUCGGUCCCTT
hsa_circ_0058495-3-F	CUGGGACCGAGGUUCAGCCTT
hsa_circ_0058495-3-R	GGCUGAACCUCGGUCCCAGTT
METTL3(H)-385-F	GCCUUAACAUUGCCCACUGTT
METTL3(H)-385-R	CAGUGGGCAAUGUUAAGGCTT
METTL3(H)-1005-F	GCUGCACUUCAGACGAAUUTT
METTL3(H)-1005-R	AAUUCGUCUGAAGUGCAGCTT
METTL3(H)-1365-F	GCUCAACAUACCCGUACUATT
METTL3(H)-1365-R	UAGUACGGGUAUGUUGAGCTT
EIF4A3(H)-369-F	GCAAUCAAGCAGAUCAUCATT
EIF4A3(H)-369-R	UGAUGAUCUGCUUGAUUGCTT

EIF4A3(H)-725-F	GGAUGAACUGAUGAAAUGTT
EIF4A3(H)-725-R	CAUUUCAUCAGCUUCAUCCTT
EIF4A3(H)-1346-F	GCAGUACUAUUCCACUCAGTT
EIF4A3(H)-1346-R	CUGAGUGGAAUAGUACUGCTT

**Biotin labeled Probe**	
has_circ_0058495-biotin	ATATAC+AGACGGC+TGAACC+TCGG+TCCCAG+AGGCTG
NC-biotin	CAGCCTC+TGGGAC+CGAGGT+TCAGCCGTC+TGTATAT

**lentivirus**	
stubRFP-sensGFP-LC3 Lentivirus	Genechem

**Kits**		
EpiQuickTM CUT$RUN m6A RNA Enrichment(MeRIP) Kit	EPIGENTEK	P-9018
ExoQuick-TCTM Exosome Precipitation Solution	SBI	EXOTC50A-1
PierceTM Magnetic RNA-Protein Pull-Down Kit	Thermo	20164
PureBinding RNA Immunoprecipitation Kit	GENESEED	P0102
PierceTM Co-immunoprecipitation Kit	Thermo	26149
RioboTM Fluorescent In Situ Hybridization Kit	RIBOBIO	C10910
Cell-LightTM EdU Apollo567 In Vitro Kit	RIBOBIO	C10310-3

**Antibody**			
Antibody name	Company	Lot	Dilution rate

IMP2(D4R2F) Rabbit mAb	CST	#14672	1:1000
Phospho-MAPK Family Antibody Sampler Kit	CST	#9910	1:1000
Rabbit Anti-eIF4A3 Polyclonal Antibody	Bioss	bs-14548R	1:1000
HA tag Rabbit PolyAb	Proteintech	51064-2-AP	1:1000
LC3A/B(D3U4C) XP Rabbit mAb	CST	#12741	1:1000
SQSTM1(H-290)	Santa Cruz	sc-26675	1:1000
Anti-β-actin (HRP-conjugate)	lifespaceo	1030300012	1:2000
DYKDDDDK tag Monoclonal antibody	Proteintech	66008-4-Ig	1:1000
TRIM25 Monoclonal antibody	Proteintech	67314-1-Ig	1:1000
Arginase-1 Polyclonal antibody	Proteintech	16001-1-AP	1:1000
iNOS Polyclonal antibody	Proteintech	18985-1-AP	1:1000
CD206 Recombinant antibody	Proteintech	81525-1-RR	1:1000
CD86 Rabbit PolyAb	Proteintech	13395-1-AP	1:1000
α Tubulin (TU-02):sc-8035	Santa Cruz	sc-8035	1:2000

The alignment of mmu_circ_0008992 and mmu_circ_0008993 with hsa_circ_0058495.

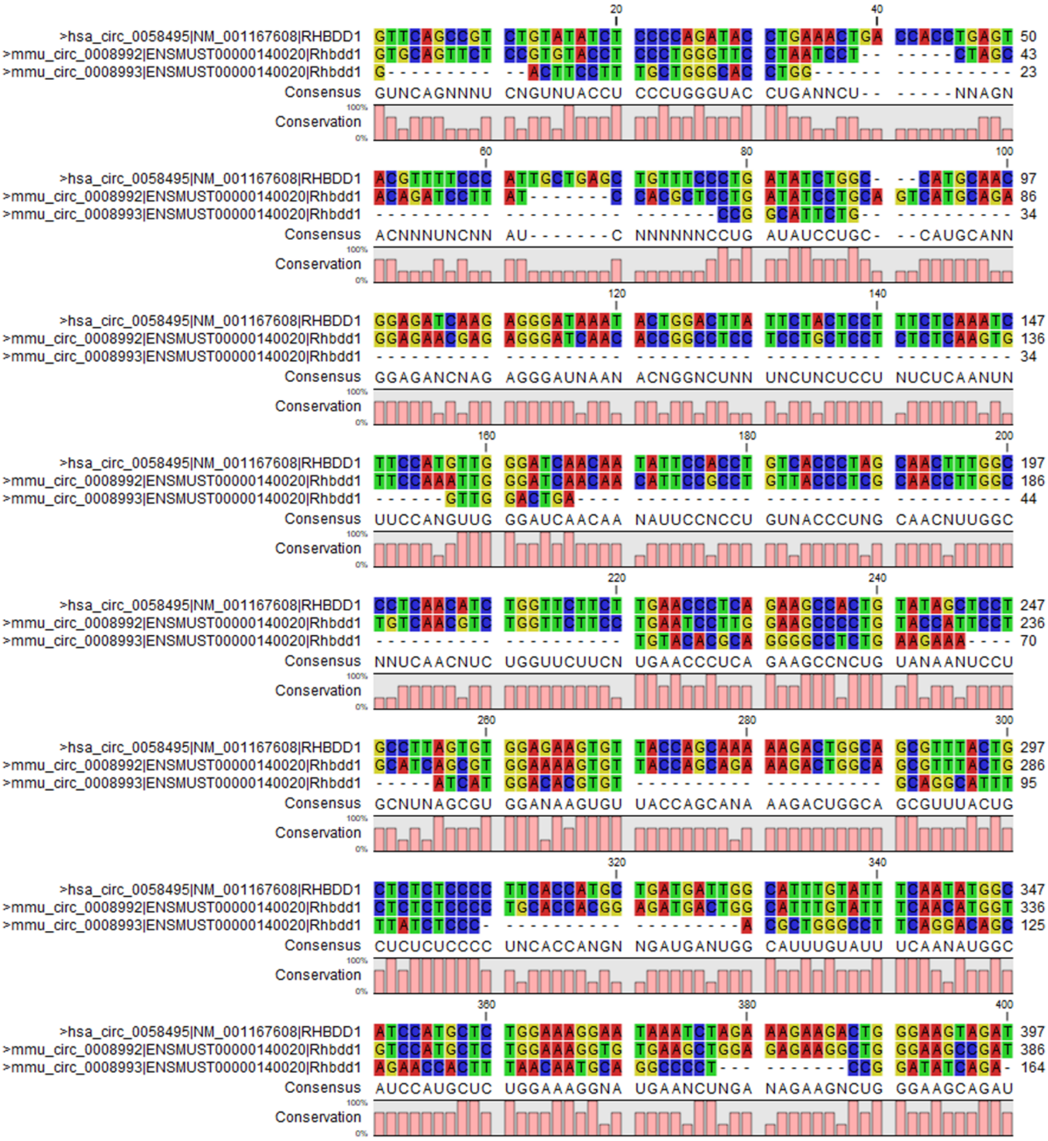

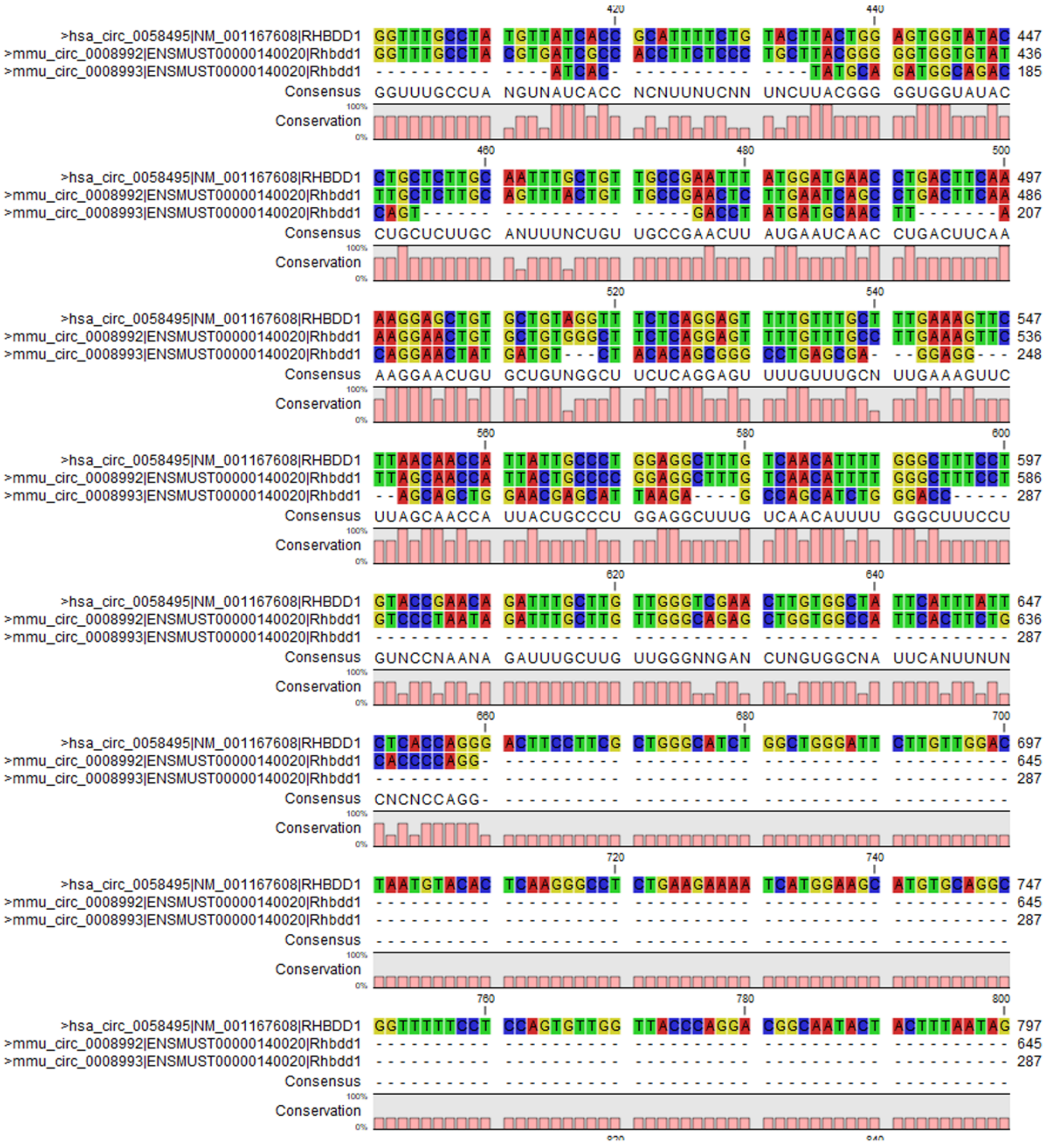

>hsa_circ_0058495|NM_001167608|RHBDD1
>mmu_circ_0008992|ENSMUST00000140020|Rhbdd1
>mmu_circ_0008993|ENSMUST00000140020|Rhbdd1
Consensus
Conservation
100%
0%

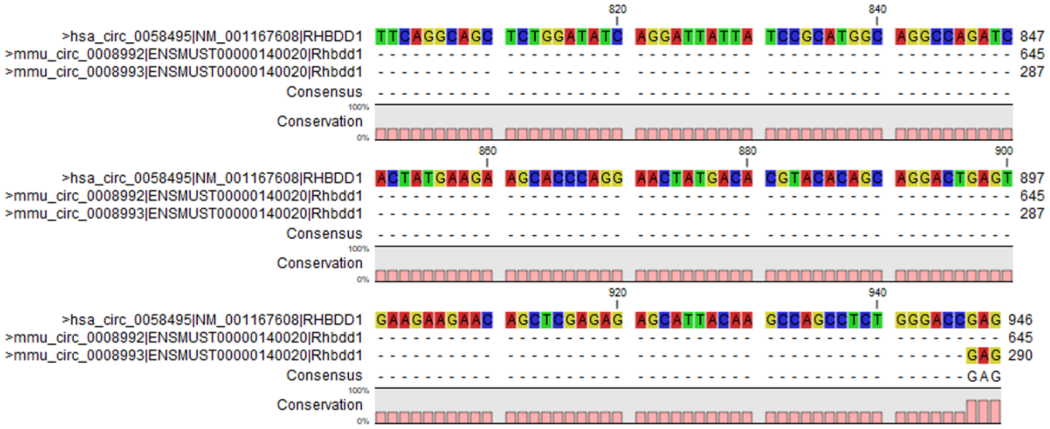

820
840
>hsa_circ_0058495|NM_001167608|RHBDD1 TTCAGGCAGC TCTGGATATC AGGATTATTA TCCGCATGGC AGGCCAGATC 847
>mmu_circ_0008992|ENSMUST00000140020|Rhbdd1 645
>mmu_circ_0008993|ENSMUST00000140020|Rhbdd1 287
Consensus
100%
Conservation
0%
860
880
900
>hsa_circ_0058495|NM_001167608|RHBDD1 ACTATGAAGA AGCACCCAGG AACTATGACA CGTACACAGC AGGACTGAGT 897
>mmu_circ_0008992|ENSMUST00000140020|Rhbdd1 645
>mmu_circ_0008993|ENSMUST00000140020|Rhbdd1 287
Consensus
100%
Conservation
0%
920
940
>hsa_circ_0058495|NM_001167608|RHBDD1 GAAGAAGAAC AGCTCGAGAG AGCATTACAA GCCAGCCTCT GGGACCGAG 946
>mmu_circ_0008992|ENSMUST00000140020|Rhbdd1 645
>mmu_circ_0008993|ENSMUST00000140020|Rhbdd1 GAG 290
Consensus GAG
100%
Conservation
0%
